# Granzyme B degrades extracellular matrix and promotes inflammation and choroidal neovascularization

**DOI:** 10.1007/s10456-024-09909-9

**Published:** 2024-03-18

**Authors:** Gideon Obasanmi, Manjosh Uppal, Jing Z. Cui, Jeanne Xi, Myeong Jin Ju, Jun Song, Eleanor To, Siqi Li, Wania Khan, Darian Cheng, John Zhu, Lyden Irani, Isa Samad, Julie Zhu, Hyung-Suk Yoo, Alexandre Aubert, Jonathan Stoddard, Martha Neuringer, David J. Granville, Joanne A. Matsubara

**Affiliations:** 1 Department of Ophthalmology and Visual Sciences, UBC, Vancouver, BC Canada; 2 School of Biomedical Engineering, UBC, Vancouver, BC Canada; 3 International Collaboration On Repair Discoveries (ICORD), Vancouver Coastal Health Research Institute, University of British Columbia (UBC), Vancouver, BC Canada; 4 Department of Pathology and Laboratory Medicine, UBC, Vancouver, BC Canada; 5 Oregon Health & Science University (OHSU), Portland, OR USA

**Keywords:** Granzyme B, Extracellular matrix, Inflammation, Angiogenesis, Age-related macular degeneration, Choroidal neovascularization, Mast cell

## Abstract

Age-related macular degeneration (AMD) is a common retinal neurodegenerative disease among the elderly. Neovascular AMD (nAMD), a leading cause of AMD-related blindness, involves choroidal neovascularization (CNV), which can be suppressed by anti-angiogenic treatments. However, current CNV treatments do not work in all nAMD patients. Here we investigate a novel target for AMD. Granzyme B (GzmB) is a serine protease that promotes aging, chronic inflammation and vascular permeability through the degradation of the extracellular matrix (ECM) and tight junctions. Extracellular GzmB is increased in retina pigment epithelium (RPE) and mast cells in the choroid of the healthy aging outer retina. It is further increased in donor eyes exhibiting features of nAMD and CNV. Here, we show in RPE-choroidal explant cultures that exogenous GzmB degrades the RPE-choroid ECM, promotes retinal/choroidal inflammation and angiogenesis while diminishing anti-angiogenic factor, thrombospondin-1 (TSP-1). The pharmacological inhibition of either GzmB or mast-cell degranulation significantly reduces choroidal angiogenesis. In line with our in vitro data, GzmB-deficiency reduces the extent of laser-induced CNV lesions and the age-related deterioration of electroretinogram (ERG) responses in mice. These findings suggest that targeting GzmB, a serine protease with no known endogenous inhibitors, may be a potential novel therapeutic approach to suppress CNV in nAMD.

**Supplementary Information:**

The online version contains supplementary material available at 10.1007/s10456-024-09909-9.

## Introduction

Age-related macular degeneration (AMD) is a multifactorial disease that is the third leading global cause of blindness [1], significantly affecting the quality of life of patients and substantially burdening the healthcare system. There are two types of AMD, the more prevalent dry (non-exudative/atrophic) AMD, which accounts for 85% of cases, is characterized by retinal pigmented epithelium (RPE) and photoreceptor loss leading to a slower, degenerative vision loss. The less prevalent wet (exudative/neovascular) AMD, which accounts for 15% of cases, surprisingly accounts for > 90% of AMD-related vision loss [2]. Wet AMD is distinguished by abnormal choroidal neovascularization (CNV), rapid subretinal or intraretinal hemorrhage, intraretinal fluid leakage into the macula, chronic inflammation and RPE detachments and thereby causes severe vision loss [2–4].

The major driver of CNV development is the potent angiogenic factor vascular endothelial growth factor (VEGF) and the current standard of care for neovascular AMD (nAMD) is intravitreal injection of anti-VEGF agents including aflibercept and ranibizumab, whilst bevacizumab is utilized as an off-label remedy [5–7]. However, anti-VEGF agents are ineffective for some nAMD patients who are either non-responsive to the treatment or develop resistance over time [8–10]. In order to improve patient outcomes, it is crucial to further comprehend the pathological mechanisms and processes of CNV closely associated with nAMD and to identify new strategies and novel targets separate from the VEGF-axis.

Here we report on targeting Granzymes (Gzms) for nAMD, a novel target that is unique and separate from the VEGF-axis. A class of serine proteases known as Gzms were first identified for their intracellular, perforin-dependent cytotoxic functions in immune cell-mediated eradication of target pathogenic cells [11, 12]. However, some Gzms also execute critical extracellular roles in immunological modulation and extracellular matrix (ECM) remodeling and vascular permeability in addition to its intracellular, pro-apoptotic functions [13]. The human genome encodes five Gzms: GzmA, B, H, K and M, with GzmB being the most understood [13, 14]. Our earlier work demonstrated that GzmB is expressed constitutively by both RPE cells and choroidal mast cells and the extracellular activity of GzmB is enhanced in choroid of older (> 65 years) compared with younger (< 55 years) eyes, and in both choroid and outer retina of nAMD eyes with CNV compared with healthy controls [15]. In this study we focus on extracellular GzmB that is produced by hyperactive, degranulating choroidal mast cells in mouse models [15]. Investigating the age-related increases in GzmB expression in the outer retina, especially the macular region, is critical to understanding the relationship between GzmB, aging and AMD. Since only nonhuman primates (NHP) have a true macula and develop drusenoid lesions comparable to humans, NHP models are therefore appropriate for such investigations before beginning clinical trials [16].

An early pathological feature of AMD is the significant remodeling of Bruch’s membrane (BrM), a critical ocular ECM which provides barrier function, support, and metabolic transport roles to the RPE and photoreceptors, regulating several signalling pathways such as angiogenesis, proliferation and survival signalling pathways [17–19]. BrM integrity has an inverse relationship with macular lesion distribution in AMD [20] and we showed that extracellular GzmB induces BrM remodeling and RPE dysfunction by cleaving important ECM proteins such as laminin and fibronectin [15]. GzmB also degrades RPE tight junctional proteins (e.g. junctional adhesion protein-A (JAM-A), zonula occludens-1 (ZO-1)) [15]. The ensuing loss of outer blood retinal barrier (oBRB) function due to RPE tight junction degradation progresses to chronic inflammation and vascular permeability [3, 21]. The chronic inflammation component of nAMD includes cytokine upregulation and leukocyte infiltration which further enhances vascular permeability and elevation of proangiogenic responses in choroidal endothelial cells [3, 22].

Consistent with our earlier work, it has been shown that in skin aging and non-ocular pathologies (including cardiovascular disease, congestive obstructive pulmonary disease, and rheumatoid arthritis), GzmB is associated with promoting pathophysiology through ECM-degradation, epithelial barrier disruption, promotion of inflammation and/or angiogenesis (for review, see Dubchak et al. [23]). Our initial work suggested that GzmB may contribute to AMD, particularly through an ECM remodelling mechanism, as many of GzmB’s substrates are present in the ECM of BrM and choroid [15]. Therefore, we hypothesize that extracellular GzmB initiates an ECM-remodeling, proinflammatory and proangiogenic pathway that triggers the development of CNV.

Here we first investigate the GzmB-aging axis in NHP and next explore the contributions of extracellular GzmB to choroidal sprouting of neovessels, cleavage of relevant ECM proteins, and subsequent release of cytokines and growth factors from the ECM that promote neovascularization. As GzmB is abundant in choroidal mast cells, we next show their choroidal distribution and the effects of mast cell degranulation/stabilization on choroidal sprouting. As our earlier work [15] demonstrated an age-dependent increase in extracellular GzmB in C57BL/6 J wild-type mice (WT) mice, we now explore the differences in retinal function and angiogenic response in GzmB-deficient mice compared to WT using in vivo imaging and ex vivo immunofluorescence. The accumulation of GzmB in the choroid of aged WT mice correlates with increased numbers of perivascular macrophages and higher levels of VEGF expression at baseline and after laser induction of CNV. ERG recordings reveal a significant age-related decrease in the a- and b-wave amplitudes in aged WT. However, in the GzmB-deficient mice, a less robust decrease in both waveforms was present and remained non-significant, suggesting an exacerbation of retinal atrophy and functional loss associated with the accumulation of extracellular GzmB in WT mice. Finally, using a specific inhibitor of GzmB, we show that the extent of choroidal sprouting is lessened. Our results support the use of GzmB inhibitors as an adjuvant treatment to suppress CNV in nAMD.

## Methods

### Animal ethics

Animals used are under the protocols approved by the University of British Columbia (UBC), Animal Care Committee, and conformed to the guidelines of the Canadian Council on Animal Care, in accordance with the Resolution on the Use of Animals in Research of the Association of Research in Vision and Ophthalmology. Laboratory mice C57BL/6 J (WT) and GzmB−/− mice with C57BL/6 J background were obtained from Jackson Laboratory (Bar Harbor, ME, USA).

### Human ethics

Human donor eyes, consented for research, were obtained from the Eye Bank of British Columbia (Canada) and were approved by the UBC Clinical Ethics Research Board and strictly adhered to the Declaration of Helsinki.

### Ocular tissue processing

Macaque, human and mouse whole globes were fixed in 10% buffered formalin or 4% paraformaldehyde, then embedded in paraffin for 6–10 µm sagittal sections. Tissue sections were mounted onto glass slides and deparaffinized using a series of alcohol washes. Some tissues underwent wholemount processing, in which eyecups were first dissected, removing the anterior segment and lens, then the neural retinas were removed to allow processing of the RPE/choroid wholemounts. For mouse eye cups, relief cuts were made to flatten the RPE/choroid tissues, then the vitreous humour removed under a dissecting microscope. Human eyecups were dissected by separating the neural retinal layer and the sclera from the RPE/choroid layers. The intact sheet of RPE/choroid was then cut into 12 equivalent pie shape pieces. Punches (3 mm in diameter) from the central, mid-peripheral and peripheral areas of each pie piece were then used for immunolabeling.

### Histological stains

#### Toluidine blue

Mast cells were identified using a 0.1% toluidine blue (Abcam, ab146366) solution in 70% ethanol/1% sodium chloride with pH adjusted to 2.0–2.5. The tissue was incubated in toluidine blue solution for 5 min, washed in PBS, then mounted using 50% Glycerol and 50% PBS, and quickly imaged at 100X (Nikon Eclipse 80i). Mast cells appeared pink/purple/red and comprised cell profiles that were approximately 10–25 µm diameter while the background was stained blue.

#### Non-specific esterase (NSE)

Mast cells and granulocytes were stained in mouse choroid cross-sections using naphthol AS-D chloroacetate (Millipore Sigma, 91C-1KT) following the manufacturer's protocol. NSE left an autofluorescent signal seen at 543 nm with confocal microscopy. Mast cells were distinguished from other granulocytes by size. NSE-positive cells were counted in central retinal regions in the mouse choroid.

### Immunohistochemistry

#### Rhesus macaque eyes

Macaque eyes were enucleated within 10 min of humane euthanasia and immersion-fixed for 24 h in 4% paraformaldehyde. The anterior chamber was removed, and the posterior eye cups were cryoprotected with 10, 20 and 30% sucrose concentrations. Samples were embedded in optimal cutting temperature (OCT) compound, frozen, and cut at 14 µm sections. Slides used for immunofluorescence staining were blocked for 30 min in a phosphate buffered saline-based buffer containing 4% horse serum, 0.5% Triton-X 100 and 1.0% bovine serum albumin. Sections were incubated with an anti-Granzyme B (Abcam, ab4059, 1:100) antibody overnight at 4 °C followed by incubation with an Alexa-Fluor 488™ secondary antibody (1:300). Slides were counterstained with 4′,6-diamidino-2-phenylindole (DAPI). Confocal z-stack images were acquired using a Leica SP5 laser-scanning confocal microscope and processed using ImageJ software.

#### Human donor eyes

Human donor eyes were processed for paraffin-embedded cross sections [15] or as RPE/choroid wholemount punches (3 mm). The samples underwent citrate buffer antigen retrieval, and then probed using primary antibody against GzmB for 2 h at room temperature (RT) and washed thoroughly with PBS, before the appropriate secondary antibody was applied and incubated for 45 min. After additional washes, the nuclei were labeled with DAPI. Next, the immunoreacted cross-sections and RPE/choroid wholemount punches were coverslipped and imaged using Zeiss LSM 800 confocal microscope at 4X, 20X, 40X or 80X magnification.

#### Mouse eyes

Mouse eye cross-sections underwent antigen retrieval with proteinase-K for 20 min or heated in citrate buffer for 10 min. A solution of 3–10% normal goat serum (NGS) (Vector Laboratories, S-1000–20) in 0.3–1.0% TX-100 in PBS was then used for 20 min to block non-specific binding. This was followed by incubation in primary and secondary antibodies (see below). In all cases, negative control sections were processed in parallel in an identical manner, except the primary antibody was omitted from the diluent and replaced with a matched IgG at the same concentration as the primary antibody.

#### Immunohistochemistry

All primary and secondary antibodies are identified in Supplementary Table 1. To characterize perivascular macrophages or GzmB-positive cells in the choroid, primary antibodies against F4/80 (Abcam, ab6640) or GzmB were used. Slides were incubated for 1–2 h at RT and left overnight at 4 °C.

#### GzmB and c-Kit double label immunohistochemistry

To determine the number of mast cells that were GzmB positive, we dissected wholemounts of mouse or human RPE/choroid for immunostaining. Following antigen retrieval with citrate buffer, the tissues were rinsed, placed in a solution of 1:100 primary antibody against c-Kit (Biolegend, 105816) for 1 h at RT, rinsed and next placed in a solution of 1:100 GzmB and incubated for 2 h at RT.

#### Angiogenic and pro-inflammation Immunohistochemistry

To assess the angiogenic potential of the outer retina of naïve and laser-induced CNV in WT and GzmB−/− mice, we used primary antibodies against CD31 (Cell Signaling 77699) or VEGF-A (Abcam, ab46154). To assess the pro-inflammatory events associated with CNV we used primary antibodies against IBA-1 (Fujifilm Wako) and IL-6 (Novus Biologicals). Each of the four antibodies was used at 1:100 dilution in 3% normal goat serum in 0.3% TX-100 PBS, and incubated at RT for 1–2 h.

After all primary antibody incubations, slides were rinsed in PBS and an appropriate fluorescently tagged secondary antibody was applied, followed by DAPI for nuclear labeling. Slides were then coverslipped and imaged.

### Confocal microscopy

Paraffin sections and wholemounts of eye tissues were imaged using a Zeiss LSM 800 confocal microscope with Zen 2.6 Blue version software (Carl Zeiss, Germany) focusing on the outer retinal areas, specifically the RPE/BrM and choroidal layers. Confocal microscopy was completed at ×20, ×40 or in some cases, a digital crop factor was applied (×80 magnification). For wholemounts, Z-stack images were taken, and orthogonal reconstructions of z-stack images were used to visualize the full depth of the wholemount, from the RPE monolayer and into the choroidal layers. Confocal images of cross-sections were taken at minimum of four positions—two central and two peripheral positions around the optic nerve. All settings on confocal were kept constant throughout the imaging sessions to compare intensity of fluorescent signals between experimental groups or between WT and GzmB−/− mice.

### Immunostaining analysis

#### CD31, F4/80 and NSE

CD31 + vessels, F4/80 + cells, or NSE + cells were identified based on their bright green (CD31 and F4/80) or their bright red (NSE) fluorescence compared to negative control sections. Positive profiles were counted on four confocal images taken in the central and mid-peripheral retinal zones. Raters of the immunolabeling were masked to the mouse strain and/or experimental group. The average number of CD31+, F4/80+, or NSE + cells per mouse was calculated and normalized by areal measurement.

#### VEGF-A analysis

Immunofluorescence of VEGF-A can be used as a relative indicator of the angiogenic potential of the mouse RPE and choroid. Images taken at identical confocal settings underwent pixel analysis using ImageJ software (NIH, Bethesda, USA). The area of interest was selected using the freehand cropping tool to quantify the mean fluorescent intensities. Threshold and radius of pixels were set to select the positively stained pixels while omitting the non-specific fluorescence artifacts. Negative control sections supported the threshold and radius settings. The histogram tool was used to count all selected pixels. Percentages of pixel counts were normalized by dividing by the total area of analysis.

### Choroid sprouting assay (CSA)

The detailed protocol of the CSA, an ex vivo model of microvascular angiogenesis is described in Shao et al. [24] Briefly, the peripheral RPE-BrM-choroid-sclera tissues were dissected into 6–10 smaller pieces of approximately 1 square mm explants from 2–3-month-old C57BL/6 J mice. The explants were placed on growth factor reduced Matrigel™ (Corning, 354,230) or Geltrex™ (ThermoFisher, A1413201) and cultured at 37 °C with 5% CO2 for 8–10 days with media changes every 48 h. For the CSAs involving exogenous GzmB treatment, CSA explants were stimulated with either exogenous GzmB (50 nM) or PBS (control) on Days 4, 6, 8 and 10 of culture. On Day 10 of culture, after five hours of GzmB treatment, culture supernatants were collected, processed, and stored at -80 °C for later analysis. For all other CSAs, on Day 2 of culture, HBSS (control), 48/80 (6.25 µg/mL), or Ketotifen Fumarate (Zaditor®, 2.0 µg/mL) was added to culture media every second day until Day 8. For those experiments using the GzmB-specific inhibitor, VTI-1002 was added once daily (250 µM). The supernatant was collected every second day and replaced by fresh media. Images of individual explants were taken every 48 h, and the areas of vascular sprouting were analyzed by a standardized SWIFT-Choroid macro based on ImageJ software and quantified relative to the original explant area or shown as total growth in pixels where 6600 pixels represents 1mm^2^ sprouting.

### Western Blot

To assess the expression and/or cleavage of various proteins after GzmB or mast cell stimulation, western blots (WBs) were performed. CSA supernatants (20–40 µl) were separated by electrophoresis on a 10% SDS-PAGE gel and transferred to a polyvinylidene difluoride (PVDF) membrane. Membranes were blocked in Blocker™ FL Fluorescent Blocking Buffer (Thermo Fisher Scientific, 37565) for 1 h at RT before probing with the following primary antibodies at a dilution of 1:1000: fibronectin (Abcam, ab2413), laminin (Abcam, ab11575), decorin (Abcam, ab175404), IL-6 (Abcam, ab6672), TGF-β (Abcam, ab92486) and VEGF-A (Abcam, ab46154) in Blocker™ FL Fluorescent Blocking Buffer overnight at 4 °C. Blots were then probed with rabbit IgG HRP-conjugated secondary antibody (R&D Systems, HAF008, 1:20,000 or 1:100,000 dilution) in 50% Blocker™ FL Fluorescent Blocking Buffer in 0.1% Tween-20-1× PBS for 2 h at RT. All membrane washes were performed with 0.1–0.2% Tween-20-1X PBS. For quality control, either the housekeeping gene, vinculin with a molecular weight of 124 kDa or total protein labeling with No-Stain™ Protein Labeling Reagent (ThermoFisher Scientific, A44449) following protocols from the manufacturer was used. The respective proteins were detected with SuperSignal™ West Pico PLUS Chemiluminescent Substrate (ThermoFisher Scientific, 34579) or SuperSignal™ West Atto Ultimate Sensitivity Substrate (ThermoFisher Scientific, A38554). Membranes were imaged with iBright FL1500 Imaging System (ThermoFisher Scientific), and densitometric band intensities relative to vinculin expression or total protein were analyzed by Image StudioLite v 5.4 software.

#### MSD U-PLEX assay

A MesoScale Discovery (MSD) U-PLEX mouse biomarker multiplex kit was used to measure concentrations of IL-6, CCL2 (also known as monocyte chemoattractant protein-1 [MCP-1]) and VEGF-A in CSA supernatant according to the manufacturer’s instructions. Supernatant samples (100 µg protein) were plated out in duplicates. The MSD 96-well plate was read on a MesoQuickPlex SQ 120 instrument within 10 min of adding the MSD read buffer. To quantify the concentrations of the analytes in the CSA supernatant, MSD.

Discover Workbench V4 software was used to generate a logarithmic standard curve according to manufacturer instructions.

### Laser-induced CNV

Two-to-12 weeks old C57BL/6J or Gzm−/− mice were anesthetized with a mixture of xylazine (6 mg/kg) and ketamine (100 mg/kg), and pupils dilated with topical drops of 2.5% Mydfrin and 1% Mydriacyl (Alcon, Fort Worth, TX). A drop of 0.5% proparacaine was used as a topical anesthetic. Next, lubricating eye drops (Alcon) were applied to the cornea. The fundus was viewed with an imaging camera, and laser photocoagulation was induced using the image-guided laser system (Micron IV, Phoenix Laboratories). The fundus image as well as the aiming beam were visualized on the monitor. Four laser burns at equal distance from the optic nerve were induced in one eye by a green Argon laser pulse with a wavelength of 532 nm, a fixed diameter of 50 μm, duration of 100 ms, and power level of 250 mW. The presence of a bubble at the time of the laser photocoagulation was considered as an indication of disruption of the Bruch’s membrane and subsequent formation of CNV.

#### Fundus fluorescein angiography (FFA)

FFA to determine choroidal neovascular leakage after laser-induced CNV was performed with the micron IV 7 days after laser photocoagulation. Mice were anesthetized, pupils dilated and intra-peritoneally injected with fluorescein AK-FLUOR (Akorn) at 5 μg/g body weight. Fluorescent fundus images were taken 10 min after fluorescein injection. The fluorescent intensity of CNV lesions was graded using ImageJ (NIH) in a masked fashion.

### In-vivo imaging

For functional in-vivo imaging, the previously developed swept source-based small animal retinal imaging system [25] was modified for a spectral domain optical coherence tomography (SD-OCT) system to achieve a better resolution and higher phase stability. A super-luminescent laser diode (BLM2-D, Superlum Diodes) with central wavelength of 810 nm and bandwidth (1/e^2^) of 90 nm was used as a light source which yields the axial resolution of 3.2 µm (in air). Polarization diversity detection for melanin-specific contrast imaging [25] was adopted by implementing two numerically calibrated custom spectrometers [26] that acquire two orthogonally polarized optical signals separated by a polarization beam splitter. The scanning beam (1 mm diameter) was directed into the pupil, with an optical power < 700 µW which is below the ANSI standard at the wavelength [27]. The OCT-based angiography (OCTA) images were derived from three consecutive OCT scanning at a single transversal location to visualize retinal vasculature. Each retinal OCT and OCTA volume data contains 500 A-scans per B-scan and 500 B-scans per volume, which were acquired at an A-scan rate of 242 kHz with a B-scan rate of 440 Hz.

The structural OCT images were constructed by averaging the two complex signals acquired by each spectrometer to form a polarization-insensitive coherent composite OCT image that compensates for the signal loss inherited from the separation of the output signals into two channels [25, 28, 29]. The vascular contrasts visualized with OCTA were obtained by conventional method of estimating complex variance between B-scans acquired at the same spot. The melanin-specific contrast images were processed by the calculation of the noise-suppressed degree of polarization uniformity (DOPU) which visualize the melanin composite in the retina [25, 29]. All computation operations for data processing and image reconstructions were conducted in MATLAB-R2022b (MathWorks).

### Electroretinograms

Two groups of mice, a younger group (2–5 months) and an older group (11–15 months) were used to compare age-related changes in ERGs and functional imaging in both WT and GzmB−/− strains. Mice were dark-adapted for 40 min prior to electroretinogram (ERG) recordings, after which all work was conducted under red light (640–700 nm). Anesthesia was administered to each mouse through an intraperitoneal (IP) injection of ketamine and xylazine. Mice remained on a temperature-controlled heating pad for the duration of the experiment. Once fully anesthetized, dilating drops (Phenylephrine 2.5%, Tropicamide 0.5% Drops) and ophthalmic anesthetic drops (Proparacaine Hydrochloride Ophthalmic Solution (Alcaine 0.5% Ophthalmic) are applied to eyes. After the eyes are checked for dilation using a red light, artificial “tear gel” (Systane Gel Lubricant) is applied to act as a buffer between the cornea and microscope lens during recordings.

Full field ERG (ffERGs) recordings were taken using the Ganzfeld ERG platform (Phoenix Laboratories). Scotopic ffERGs were recorded at seven intensities ranging from − 1.7 to 2.1 log (cd s/m^2^) with 10–20 flash stimuli presented at each intensity. Special care was given to increase time intervals and lowering flash duration as stimuli intensity increased to prevent photoreceptor bleaching. Recordings were taken from both eyes. ERGs from the right eye were recorded for 300 ms after the flash stimuli, producing both a and b-waves. Left eye ERGs recorded for 2 s after each flash to account for the c-wave as well. All data were collected and analyzed in LabScribe Data Recording Software (iWorx). Later, mice were injected with a reversal agent atipamezole hydrochloride (ANTISEDAN® Zoetic Inc, 1 mg/kg) or 0.9% saline (20 ml/kg) subcutaneously and placed on heating pads until fully awake and returned to cages.

### Statistics

Statistical data were analyzed using GraphPad Prism version 9 and expressed as mean ± standard error of the mean. Analysis between two groups of continuous variables was conducted using independent samples t-test while analysis between multiple groups of continuous variables was conducted using one-way ANOVA followed by a Tukey’s post-hoc test. Analysis between two groups of ordinal variables was conducted with a one-tailed Mann–Whitney U test. A p-value < 0.05 was considered statistically significant.

## Results

### GzmB immunoreactivity in NHP outer retina

**Fig. 1:**
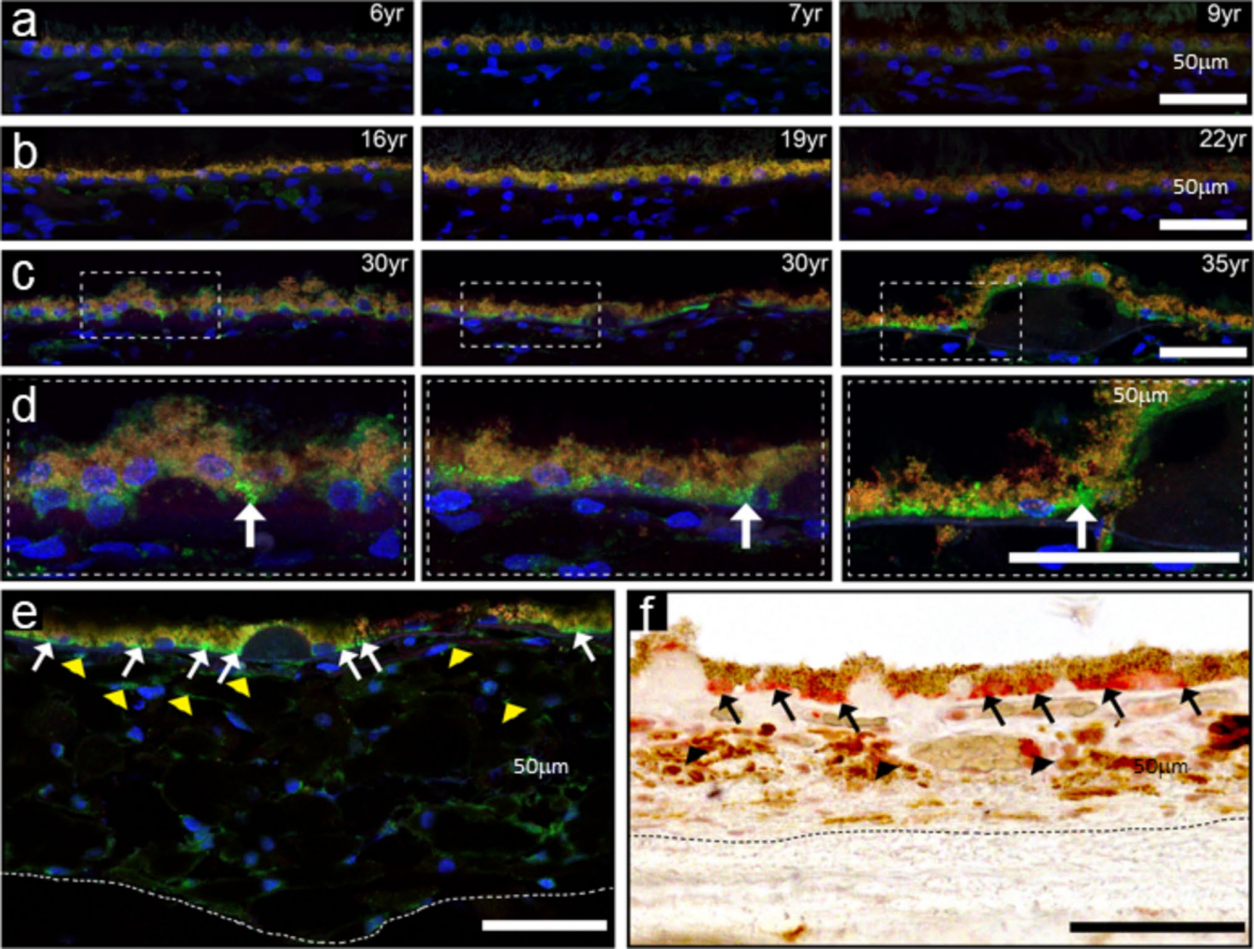
Comparison of GzmB immunoreactivity in primate and human outer retina. **A**–**C** Cross sections of RPE and choroid from young (**A**), middle aged (**B**) and older (**C**) rhesus monkey demonstrate age-related increase in GzmB immunofluorescence (green) in the basal compartment of the RPE in 30–35 year old rhesus surrounding drusen sites. Enlargement of dashed boxes shown in (**D**). **D–E** GzmB immunoreactivity in 35 year old rhesus with GzmB immunofluorescence evident in RPE (white arrows) and in choroid (yellow arrowheads). **F** A 70-year old human donor eye demonstrates GzmB immunolabeling in basal compartment of RPE (AEC red chromogenic label, black arrows) surrounding soft drusen indicating similarity between rhesus and human eye tissues. The choroidal mast cells (black arrowheads) are also immunoreactive for GzmB. Dotted line demarcates choroidal/scleral interface

The nonhuman primate (NHP) is a relevant animal model for undertaking experimental studies for new treatment strategies for AMD [30, 31]. Here, we asked whether GzmB distributions in the NHP retina mirror those observed in the human retina from our earlier study [15]. We grouped NHP (rhesus macaques) into younger (Fig. 1A), middle-aged (Fig. 1B), and older (Fig. 1C, D) age categories and determined that the older NHP and the older human retina have similar distribution patterns of GzmB immunoreactivity which includes the ECM of BrM, the basal compartment of the RPE (particularly near drusen sites) and choroidal immune cells (Fig. 1E, F). The NHP data allowed us to probe younger eyes, not available in the post-mortem human study, to confirm that the immunoreactivity for GzmB increases with age in NHP from 6 to 35 years of age.

### Exogenous GzmB promotes vascular sprouting and VEGF-A expression while reducing TSP-1 in the RPE-choroid

**Fig. 2:**
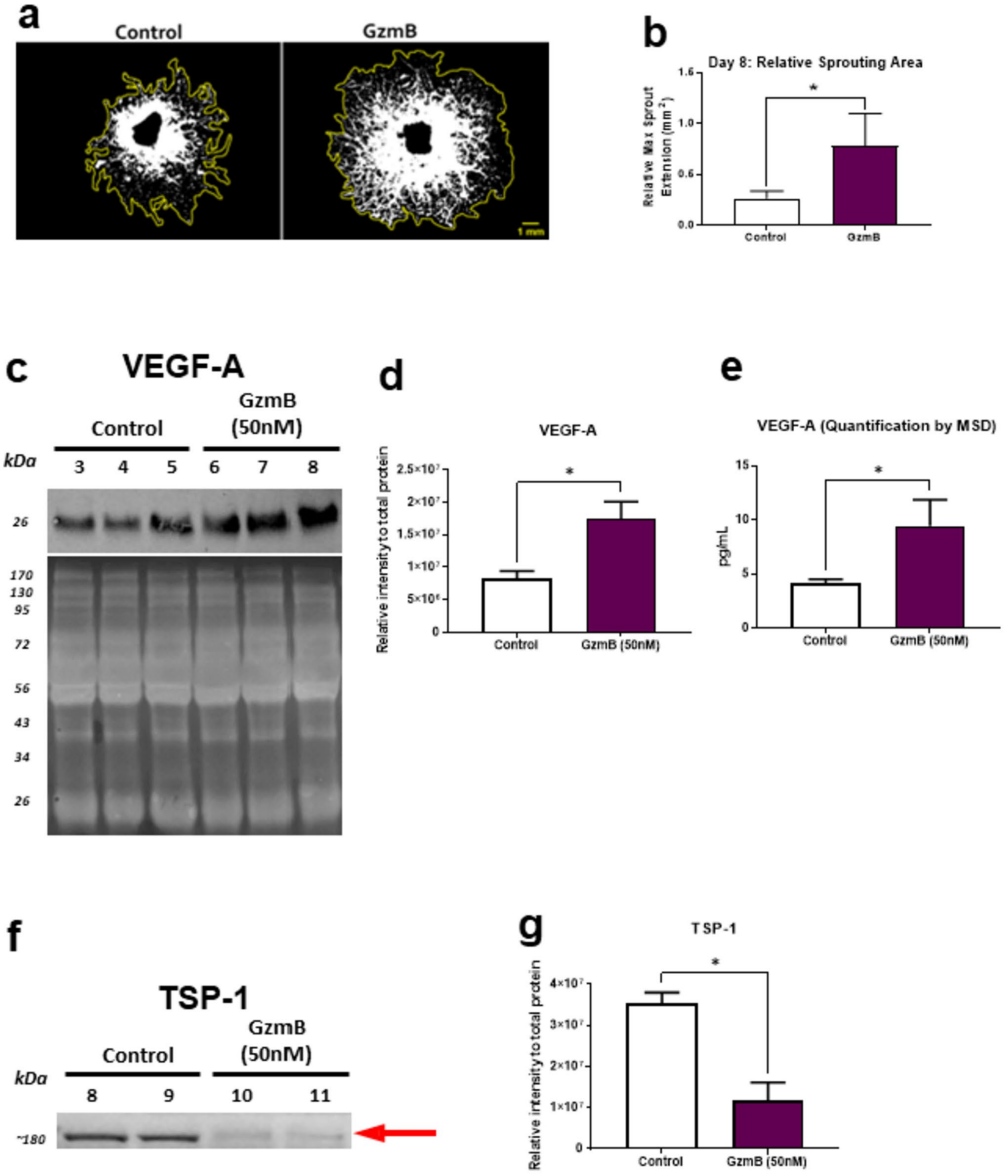
GzmB promotes vascular sprouting and VEGF-A expression while reducing TSP-1 in the RPE-choroid. **A** Representative images show the effects of exogenous GzmB treatment compared with controls at Day 8 of explant culture. Yellow lines around sprouting area show maximum extent of sprouting. **B** Quantification of vascular sprouting at Day 8 of CSA. Choroid sprouting is significantly increased with GzmB treatment compared with control (PBS) treatment. **C**–**E** Western blot and MesoScale Discovery (MSD) multiplex assays reveal GzmB-induced increased expression of VEGF-A in CSA supernatant. **C** Representative western blot of VEGF-A and its total protein control blot for total protein normalization (iBright Imaging Systems) **D** Densitometric quantification of VEGF-A in western blot. **E** Quantification of VEGF-A in MSD multiplex assays. **F**–**G** Western blot assays reveal GzmB-induced proteolysis of TSP-1 in CSA supernatant. The total protein control blot is shown in Supplementary Fig. 1. **F** Representative western blot of TSP-1. **G** Densitometric quantification of TSP-1 western blot. Results are presented as mean ± SEM. *p < 0.05 in T-test. n of A–D, F and G = 6 per group, n of E = 4–6 per group

The proangiogenic effects of exogenous GzmB in the choroidal sprouting assay (CSA) were investigated [32]. After 10 days of explant culture, GzmB-treated and control explants both showed a flurry of choroidal vascular sprouting (Fig. 2A). However, compared with controls, GzmB-treated explants demonstrated significantly increased choroidal vascular sprouting (Fig. 2B).

To verify whether VEGF-A expression is altered in the GzmB-treated tissue with enhanced vascular sprouting, VEGF-A levels in CSA supernatant were measured with western blot (WB) and Meso Scale Discovery (MSD). MSD, a sensitive electrochemiluminescence-based assay is ideal for detecting multiple low-abundance proteins like growth factors and cytokines in a single sample. This method complements our WB findings. WB revealed increased expression of VEGF-A in the GzmB-treated CSA supernatant compared to controls in the WB (Fig. 2D) and MSD (Fig. 2E) experiments. Furthermore, on measuring TSP-1, a known anti-angiogenic factor, with WB in the CSA supernatant, there was a significantly reduced expression of TSP-1 in the GzmB-treated CSA compared with controls (Fig. 2F–G, Supplementary Fig. 1).

### Exogenous GzmB degrades the extracellular matrix and promotes inflammation in the RPE-Choroid

**Fig. 3:**
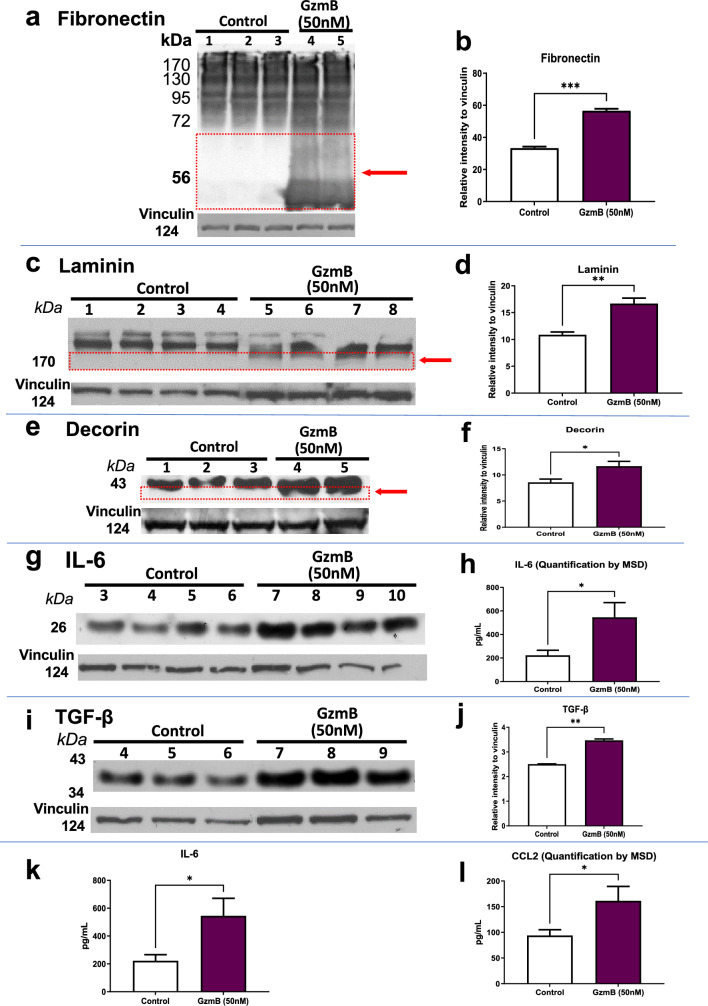
GzmB degrades the extracellular matrix and promotes inflammation in the RPE-Choroid. **A**, **C**, **E** Western blot reveals cleavage of extracellular matrix proteins by exogenous GzmB. Representative western blot of ECM proteins in CSA supernatant for **A** fibronectin; **C** laminin and **E:** decorin. Note cleavage bands at lower molecular weight, identified by the red box and arrow in A (fibronectin) and C (laminin). Vinculin bands are shown as loading controls. **B**, **D**, **F** Densitometric quantification of degradation by western blot—the additional cleavage bands at lower molecular weight were quantified. **B** Fibronectin; **D** laminin and **F** decorin. Results are presented as mean ± SEM. *p < 0.05, ***p < 0.001 in T-test. n = 4 per group. **G**, **I** Next, we tested pro-inflammatory cytokines by western blot in CSA supernatant after exogenous GzmB. Representative western blot of inflammatory cytokines in CSA supernatant: **G** IL-6; **I** TGF-β. **H**, **J** Densitometric quantification of western blots. **H** IL-6; **J** TGF-β**. K**, **L** Two additional pro-inflammatory cytokines were quantified by MSD multiplex assay: **K** IL-6; **L** CCL2. Results are presented as mean ± SEM. *p < 0.05, **p < 0.01, ***p < 0.001 in T-test. n = 4–6 per group

To verify whether GzmB can degrade relevant ECM proteins in the RPE-Choroid such as fibronectin, laminin and decorin, exogenous GzmB was added to ex vivo CSA explants. WB revealed degradation of fibronectin (Fig. 3A), laminin (Fig. 3C) and decorin (Fig. 3E) in the supernatant of the CSA after GzmB stimulation. Densitometric analysis shows that degradation of fibronectin (Fig. 3B), laminin (Fig. 3D) and decorin (Fig. 3F) were significantly higher in samples stimulated with GzmB compared with controls.

In previous studies, we have shown that GzmB accumulation is associated with chronic inflammation in cardiovascular, pulmonary and skin pathologies [13, 33–35]. Here we assessed inflammatory cytokines and growth factor expression after GzmB stimulation in the CSA. WB revealed increased expression of IL-6 (Fig. 3G) and TGF-β (Fig. 3I) in the CSA supernatant after GzmB stimulation. Densitometric analysis shows that IL-6 (Fig. 3H) and TGF-β (Fig. 3J) were significantly higher in samples stimulated with GzmB compared with controls. Furthermore, MSD also revealed significantly increased expression of IL-6 (Fig. 3K) and CCL2 (Fig. 3L) in the GzmB-treated CSA supernatant compared with controls.

### GzmB deficiency suppresses VEGF and macrophages in outer retina

**Fig. 4:**
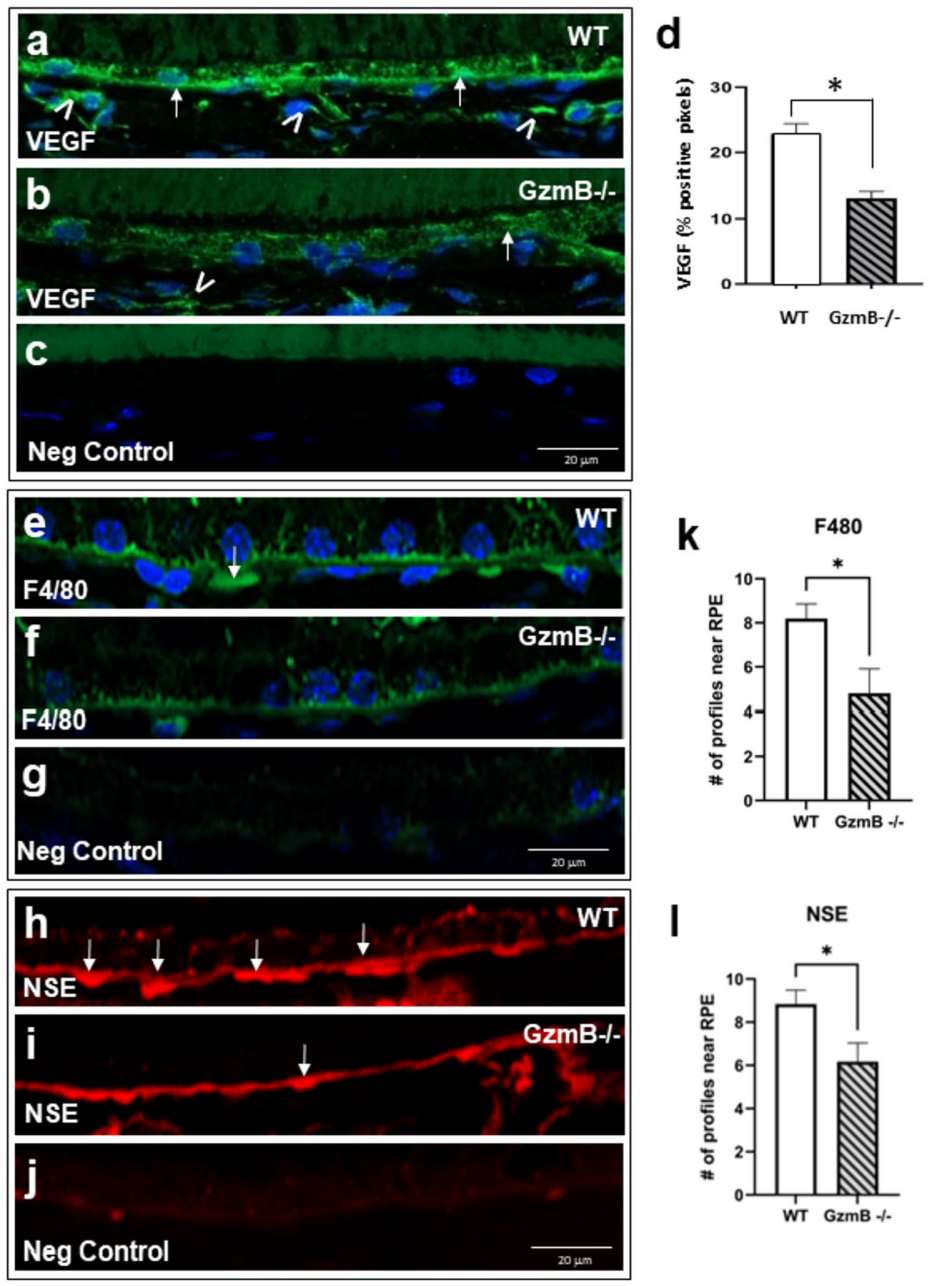
GzmB deficiency reduces angiogenic growth factor, VEGF, and macrophages in outer retina. **A–C** Vascular endothelial growth factor (VEGF) immunofluorescence is robust in outer retina of WT but dramatically reduced in the GzmB−/−. **A**, **B** VEGF immunofluorescence (*IF*) reveals strongly labeled RPE (arrows) and endothelial cells in walls of vessels (arrowheads) in WT compared to GzmB−/−. **C** Negative control demonstrates the specificity of the *IF* in which the primary VEGF antibody was omitted and replaced with a non-immune antibody of the same isotype, while keeping all other steps identical during processing. **D** Average percentage of pixels positive for VEGF immunofluorescence is significantly greater in WT compared to GzmB−/− (n = 4, * p < 0.05, Mann–Whitney U Test). **E**, **F** Perivascular macrophage markers F4/80 immuno-fluorescence (green, arrow) is significantly more robust in outer retina of WT vs. GzmB−/−. **G** Negative control demonstrates the specificity of the *IF* in which the primary F4/80 antibody was omitted and replaced with a non-immune antibody of the same isotype, while keeping all other steps identical during processing **H**, **I** NSE *IF* (red, arrows), labels macrophages and is significantly more robust in outer retina of WT vs GzmB−/−. **J** Negative control demonstrates the specificity of the *IF* in which the NSE reagent was omitted and replaced while keeping all other steps identical during processing. **K**–**L** Number of labeled profiles is significantly greater in WT compared to GzmB−/− (n = 4, * = p < 0.05, Mann–Whitney U Test). All tissue samples are from 9-month-old WT or GzmB−/− mice

Given that extracellular GzmB accumulates in WT mice with age, we asked whether aged GzmB−/− mice on the same C57BL/6J background would demonstrate a lower angiogenic potential, as our hypothesis is that extracellular GzmB initiates ECM-remodeling and proangiogenic pathways that trigger the development of CNV. We probed cross-sections of outer retina for VEGF and macrophages. A significant increase in VEGF immunofluorescence was observed in BrM, RPE and endothelial cells in the vascular walls was observed in the choroid in the WT compared to the GzmB−/− (Fig. 4A-D). Perivascular macrophages were evident and more numerous as shown by F4/80 and non-specific esterase (NSE) in the WT compared to the GzmB−/− (Fig. 4E–L).

### GzmB deficiency improves ERG responses and minimizes the angiogenic and pro-inflammatory response after laser-induced CNV.

**Fig. 5:**
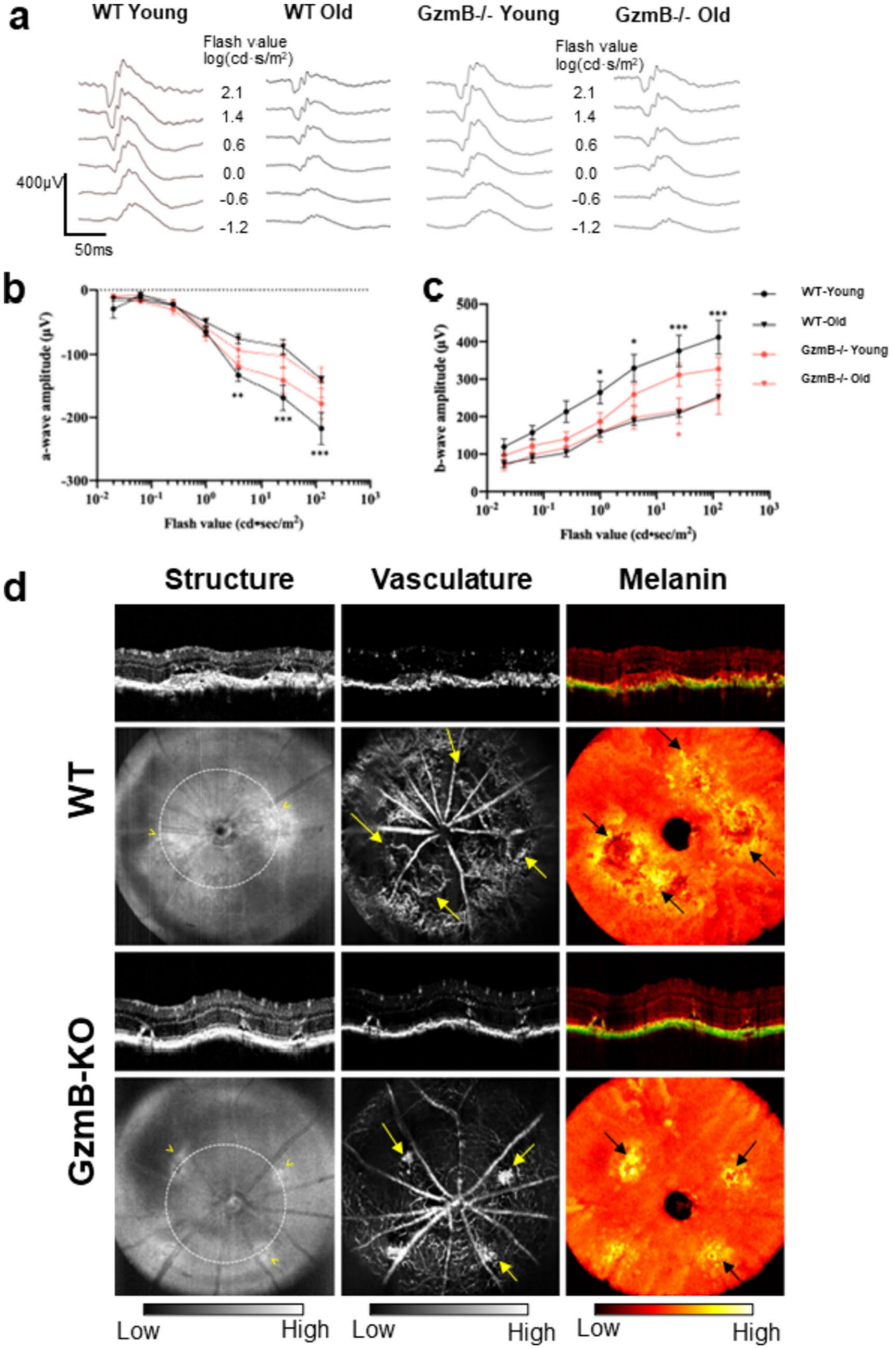
GzmB deficiency improves ERG and minimizes laser-induced CNV. ERG a- and b-wave amplitude significantly decrease with aging in the WT, while the age-related alterations in ERGs in GzmB−/− mice are not significant**. A** Representative images of scotopic ff-ERG wave form in young (2 to 5-month-old) and old (11 to 15-month-old) C57BL/6 J WT and GzmB−/− mouse respectively. **B** Significant a-wave amplitude was observed in WT young vs. old in flash value 0.6, 1.4 and 2.1 log(cds/m^2^), while changes in GzmB−/− young vs. old mice were not significant. **C** Significant b-wave amplitude was observed in WT young vs. old in flash value 0, 0.6, 1.4 and 2.1 log (cds/m^2^), while only slight differences were detected in GzmB−/− young vs. old mice at flash value of 1.4 log(cds/m^2^). *p < 0.05, **p < 0.01, ***p < 0.001, mean ± SEM. N = 4–5 per strain and age groups. **D** In vivo retinal OCT imaging at Day 7 post laser induction demonstrates reduced CNV lesions in the GzmB−/− compared to WT. Multi-contrast en-face projection images of the CNV lesions in a WT retina demonstrated large structural lesions (yellow arrowheads) with large vascular loops (yellow arrows) and elevated melanin-containing regions (black arrows). Laser-induced CNV lesions in the GzmB−/− retina were smaller structural lesions (yellow arrowheads) that are clearly delineated by melanin contrast displaying focal elevated melanin-containing regions (black arrow), with a confined vascular extent (yellow arrows), which are indicative of a lower angiogenic response associated with the laser induction. Circularly projected multi-contrast cross-sectional images of WT and GzmB−/− retina were extracted from the white dotted lines marked at structural *en-face* projection images

The ERG is a non-invasive method to measure visual function to assess age-related changes, progression of eye disease and retinal degeneration [36]. As the WT mouse accumulates extracellular GzmB with age, while the knockout does not, we asked whether the lack of GzmB would ameliorate the age-related deterioration in the a-wave and b-wave amplitudes previously shown in C57BL/6J and other strains of mice [37, 38]. Our results on younger (< 5 months) and older (> 12 months) WT mice confirmed a significant age-related decrease in both a- and b-wave amplitudes at higher flash intensities; however, as predicted there were minimal age-related changes in the amplitudes in the GzmB−/− mice (Fig. 5A–C).

**Fig. 6:**
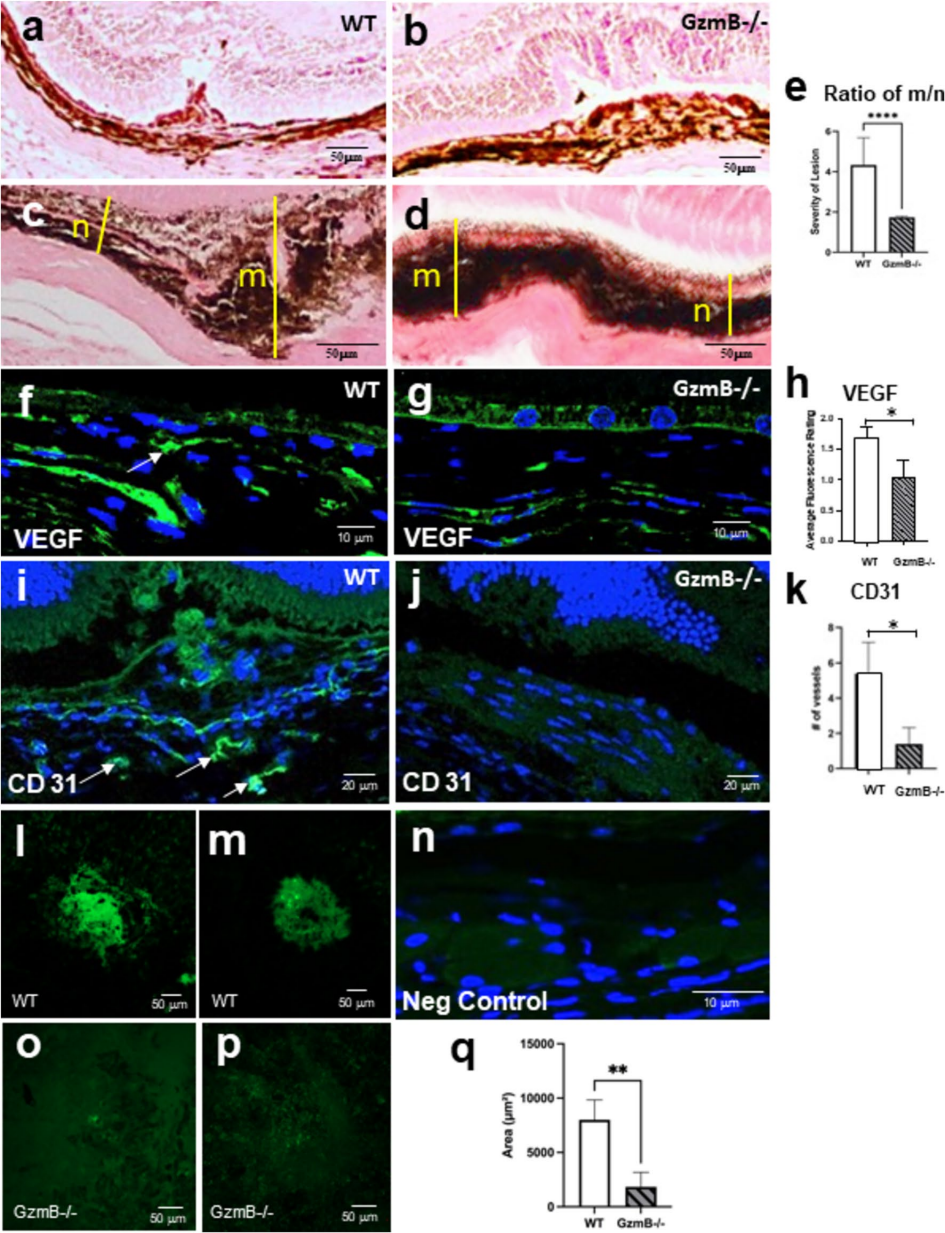
GzmB deficiency suppresses VEGF and neovascularization after laser induction. **A**–**E** Hematoxylin-stained cross-sections through the center of CNV lesion in WT (**A**, **C**) and GzmB−/− mouse (**B**, **D**). CNV lesions in WT mouse extend into the ONL and INL, while lesions in the GzmB−/− mouse extend in the ONL, but is less disruptive of the neuroretinal layers. Quantification of the CNV was undertaken by measuring the thickness within the center of the CNV lesion (m) and compared to the thickness of the adjacent, intact, unaffected choroidal and RPE layer (n). The ratio of m/n demonstrates that the CNV lesions in the WT were significantly more robust than those in the GzmB−/− mice (**E**). **F**–**H**: Confocal images of VEGF-A *IF* (green) adjacent to the CNV lesion reveals stronger *IF* in choroid of WT (**F**) compared to GzmB−/− (**G**) (arrow). **H** Average fluorescence rating of VEGF *IF* is higher in WT compared to GzmB-KO. p < 0.05 N = 5; Mann–Whitney U, one tailed). **I-K**: CD31 *IF* reveals strongly labeled endothelial cells in walls of vessels (arrows) after CNV induction in WT (**I**), lower *IF* in GzmB−/− mice (**J**) after CNV induction. **K** Average number of vessels/cross section positive for CD31 in significantly greater in WT vs GzmB−/−. p < 0.05. n = 5 Mann–Whitney U, one tailed. **L**–**M** Fluorescent image of IB-4 (for proliferating endothelial cells) in choroid flatmounts after laser-induced CNV in WT. Note large extent of CNV membrane as indicated by green fluorescence when compared to images in GzmB−/− after CNV induction (**O**–**P**). **Q**: Areal measurements reveal a significant reduction of CNV membrane growth in GzmB−/− mice; p < 0.01 n = 3 GzmB−/− and n = 5 WT

In vivo imaging was performed using a custom-built optical coherence tomography (OCT) specifically designed for high-resolution mouse retinal imaging [39]. In addition, polarization diversity detection was employed into the system for extracting melanin-specific contrast, and multi-contrast acquisition and processing algorithm were utilized based on the previous work [25]. Using the high-resolution and multi-contrast OCT system, the distinctive characteristics in structural, vascular, and melanin distribution aspects of CNV development between WT and GzmB−/− mice were demonstrated. Figure 5D shows representative in vivo mouse imaging results selected from WT (n = 13) and GzmB−/− (n = 14), which emphasizes noticeable variations in CNV lesion size, angiogenic response, and melanin contents associated with the deficiency of GzmB. In particular, the laser-induced CNV lesion were dramatically smaller with less melanin content in the GzmB−/− compared to WT mice, and a reduced neovascularization as demonstrated by vascular contrast, confirming a lower angiogenic response associated with the deficiency of GzmB (Fig. 5D). We further explored the in vivo findings by immunohistology and quantified CNV in ex vivo tissues. Given that GzmB deficiency resulted in lowered levels of VEGF and perivascular macrophages in the outer retinal of the GzmB−/− mouse (Fig. 4), we next asked whether these changes would affect CNV development after laser induction. Seven days post laser induction, animals were sacrificed and CNV lesions were quantified. In histological cross-sections through the peak of the lesion, the WT lesions formed a peaked proliferative growth in the RPE and choroidal layers (Fig. 6A, C) whereas the lesions in the GzmB−/− displayed a shallower (less peaked) growth (Fig. 6B, D). The thickness from the bottom of the pigmented choroid to the top of the neovascular membrane (m) was compared to the thickness of the intact-pigmented choroid and RPE adjacent to the lesion (n) to estimate the size of the lesion in the cross-sectional plane (ratio m/n) to demonstrate that the lesions in the GzmB−/− were significantly smaller than those in the WT (Fig. 6E). Furthermore, the immunoreactivity for two indicators of an angiogenic response, VEGF and CD31, a proliferating endothelial cell marker, also demonstrated that GzmB−/− mice mounted a significantly lower angiogenic response and lesions that were smaller in areal measurements than those observed in WT (Fig. 6L–Q).

**Fig. 7:**
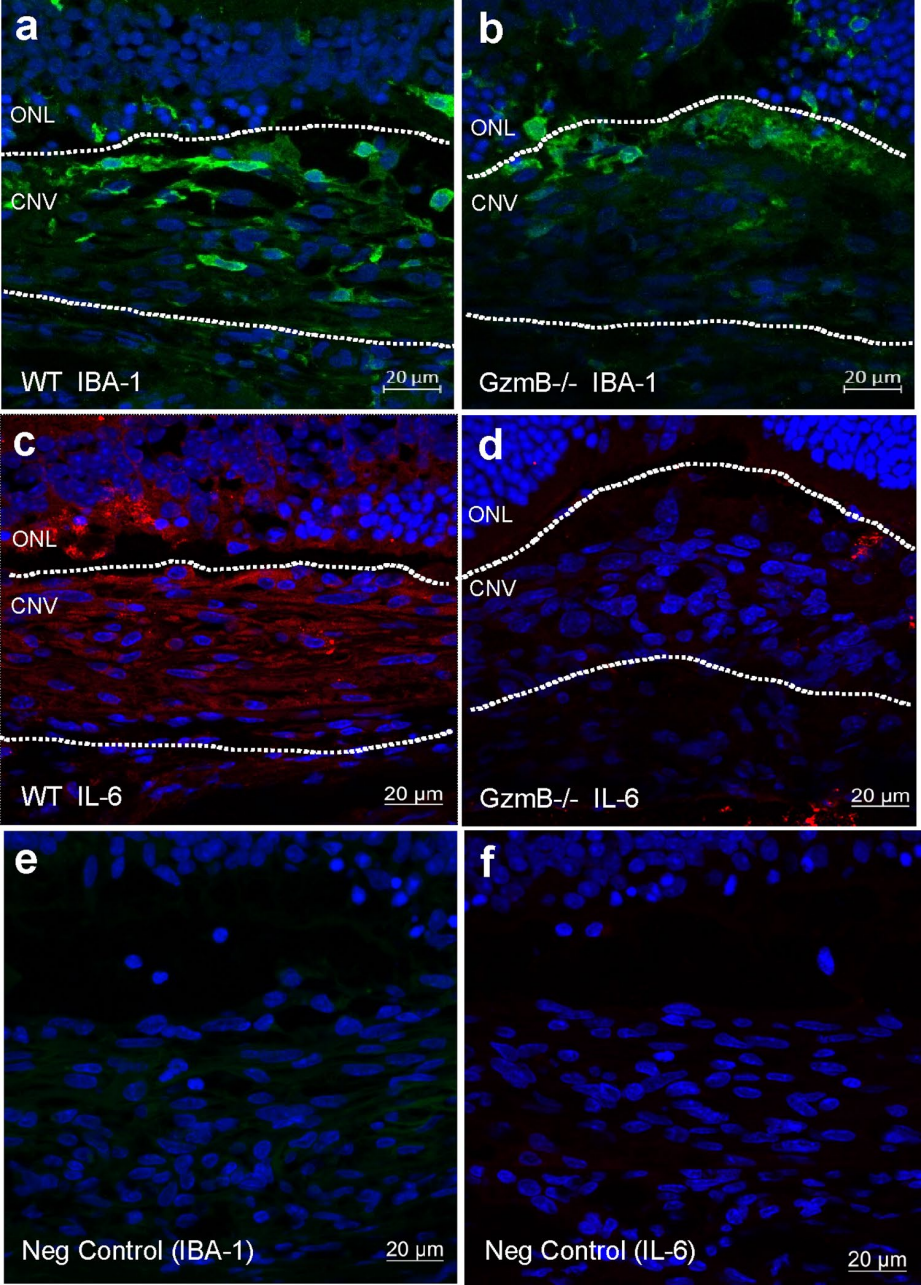
GzmB deficiency suppresses inflammation after laser induction. **A-D**: Cross-sections through CNV lesions (white outlines) in WT (**A**, **C**) and GzmB−/− (**B**, **D**). Cross sections were immunoreacted for IBA-1 with a 488 nm labeled secondary antibody (green; **A**, **B**) or IL-6 with a 546 nm labeled secondary antibody (red; **C**, **D**). Note that in the WT, the immunofluorescence associated with IBA-1 (a microglia marker) and IL-6 (pro-inflammatory cytokine associated with CNV) is greater than seen in the GzmB−/−. Negative control (**E**, **F**) images reveal a lack of non-specific immunofluorescence. *ONL* outer nuclear layer, *CNV* choroidal neovascular lesion

The angiogenic response observed in the WT was mirrored by an inflammatory response using two indicators of inflammation, IBA-1 (a marker of microglia, a resident immune cell in the retina) and IL-6 (a cytokine associated with vascular permeability and chronic inflammatory responses in retinal tissues). Immunofluorescence levels were higher in the WT compared to the GzmB−/− mice for IBA-1 (Fig. 7A, B) and IL-6 (Fig. 7C, D), with negative controls demonstrating a lack of non-specific labeling (Fig. 7E, F).

### Characterization of GzmB-expressing mast cells in the human and mouse choroid/sclera

**Fig. 8:**
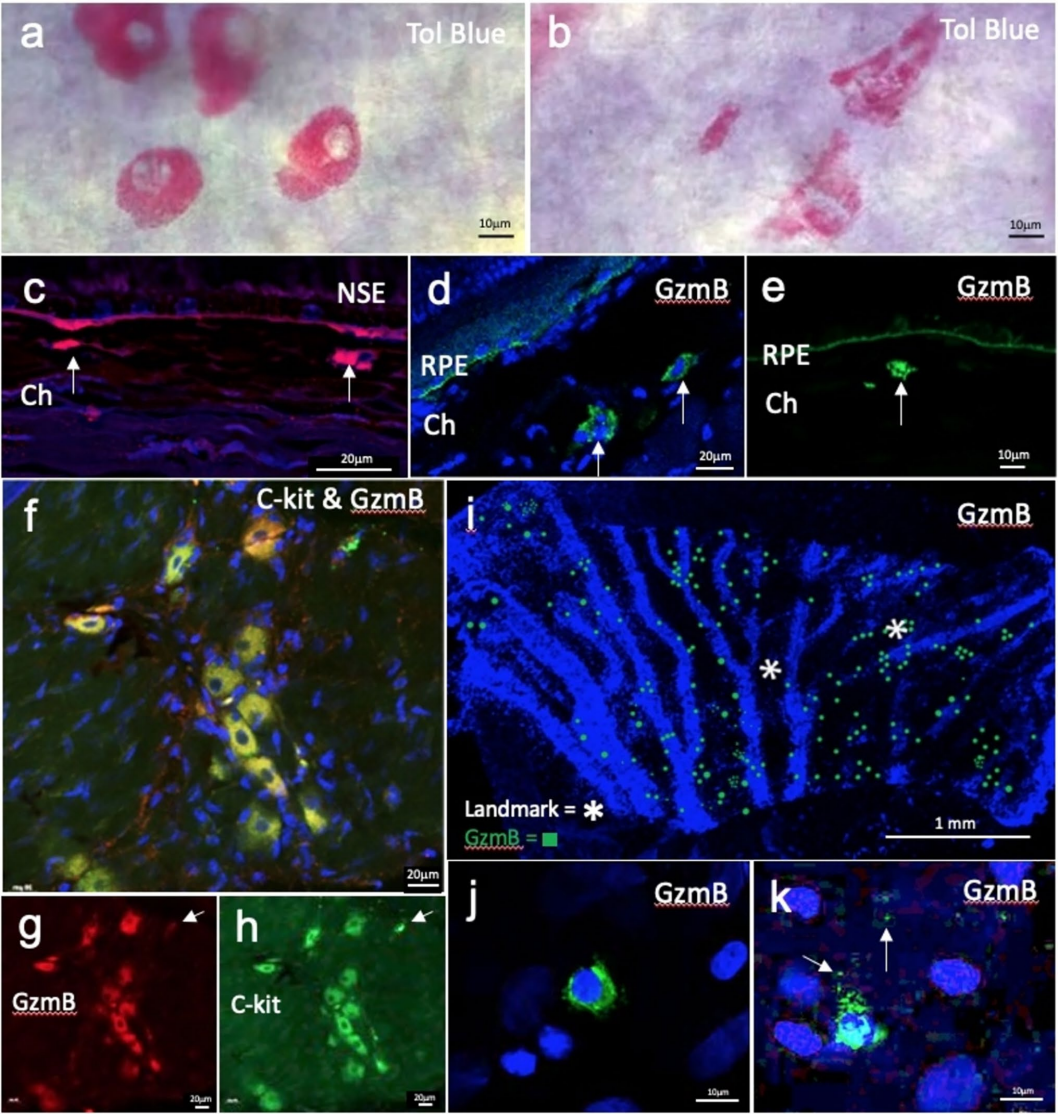
Mast cells stain positive for Toluidine blue, NSE, GzmB, and C-kit in mouse choroid/sclera and positive for GzmB in human. **A** Representative image showing the presence of toluidine blue positive mast cells (in pink) in an albino mouse choroidal wholemount at 100X magnification. **B** Representative image revealing mast cells in the process of degranulation in an albino mouse choroidal wholemount. **C** Image showing the presence of NSE positive cells in the choroid of a mouse choroid cross section at 40X magnification. Red color represents NSE, a marker of mast cells and macrophages and the blue color is DAPI [43, 44]. Arrows point to NSE label that is from Mast cells. **D** Showcases a granular GzmB positive cell in a mouse choroid/sclera cross-section. Arrow points to an example cell at 20X magnification. **E** Representative image of a mast cell positive for GzmB in the upper choroid in mouse. **F**–**H** Double label image of C-kit (green) and GzmB (red) in mouse choroid wholemount showing mast cells at 20X magnification. While the majority of the cells co-label GzmB and C-kit, the arrow points to a C-kit only cell, suggesting different populations of mast cells based on types of proteases. Asterisk highlights extracellular GzmB in choroid. **I** Human choroidal wholemount pie piece labelled with GzmB showing relative distribution to choroidal vasculature. **J**–**K** Representative image showcasing non-dengranulated and degranulating mast cells in Human choroidal wholemount. Scale bar in **A**, **B**, **E**, **J**–**K** is 10um. Scale bar in **C**, **D**, **F**–**H** is 20um. Scale bar in I is 1 mm

Next, we asked what is the contribution of mast cells to CNV, as our earlier work demonstrated that choroidal mast cells, in addition to RPE cells, contain GzmB [15]. Earlier work investigating the role of choroidal mast cells in AMD focused on rat models. To date, limited data exist for mice [40, 41]. To confirm the presence of mast cells in the mouse choroid, we conducted toluidine blue staining, a well-known method used to identify mast cells. Toluidine blue^+^ profiles in the mouse choroid wholemount ranged from dark purple to bright pink in color. On average, mast cell diameters ranged from 10µm to 25µm, while some had a dispersed appearance with a diameter over 30µm. At higher resolutions (100X), as shown in Fig. 8A, the toluidine blue profiles had a granular appearance wherein individual granules could be identified. The color, size, location and granular appearance helped us identify mast cells. Despite the absence of known mast cell activators, some cells appeared to be actively degranulating, as shown in (Fig. 8B). We supplemented the toluidine blue data with mouse choroidal cross-sections in Fig. 8C, which show NSE^+^ mast cells in wildtype mice. NSE labels cells of the granulocytic lineage and macrophages. NSE^+^ profiles in the choroid ranged from 10-20µm, similar to the size seen in the wholemounts in Fig. 8A. These profiles did not have a granular appearance which is likely caused by the lower magnification of the image and the autofluorescent nature of the NSE stain.

Previously, while investigating GzmB in mouse outer retina, we observed that GzmB accumulation also occurs in aging mice. However, GzmB was primarily detected proximal to the BrM/ RPE [15]**.** To confirm that GzmB^+^ cells also exist in mouse choroid, we labelled cross-sections for GzmB. Although intense GzmB positivity proximal to the BrM, as reported previously [15], Fig. 8C and 8D also illustrate evidence of GzmB-positive mast cells in the choroid [15]. These mast cells are observed at various depths in the mouse choroid, similar to what was reported on human choroidal mast cells [42]. After subsequent immunostaining, we observed that the GzmB signal from these cells co-localizes with c-Kit, a prominent mast cell marker in mouse [43]. Figure 8 F–H reveal examples of c-Kit^+^ mast cells in mouse choroidal wholemounts that contain GzmB.

From comparing Fig. 8G and H, we noted that not all c-Kit^+^ cells were GzmB^+^ (arrow), suggesting that there may be sub-populations of mouse mast cells expressing various levels of GzmB or other proteases.

### Human choroidal mast cells are GzmB^+^ and localize near blood vessels

Human choroid in cross sections also contains GzmB^+^ mast cells as we showed earlier [15]. Here we explored GzmB^+^ profiles in a human choroidal wholemount. Figure 8I reveals the distribution of mast cells positive for GzmB, where mast cells had distinct cytoplasmic granules and ranged from 10-25 µm in diameter. GzmB^+^ mast cells localized near blood vessels and had a distinct pattern of clustering in some choroidal areas, pattern of clustering in some choroidal areas, while being vacant in others, as shown on the distribution map in Fig. 8I. Some mast cells were imaged at higher resolution and seen in a non-activated state (Fig. 8J); however, mast cells were imaged in a degranulated state with immunofluorescence indicating GzmB granules in the extracellular spaces in the choroid (Fig. 8K).

### Mast cell degranulation and extracellular GzmB contribute to choroidal sprouting

Although mast cells contain many different components ranging from growth factors, cytokines, amines and proteases, they have been reported to have a net proangiogenic effect [42, 45–47].

**Fig. 9:**
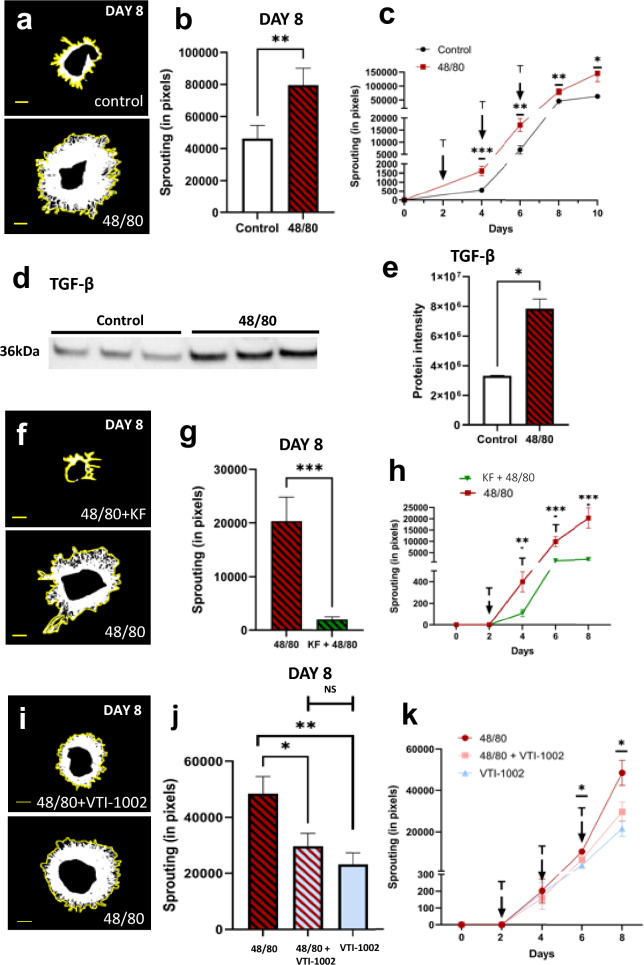
Inhibition of choroidal mast cell degranulation suppresses choroidal angiogenesis. (**A**) Representative images showing the effects of 48/80 treatment compared to control at Day 8 of explant culture. Yellow lines outline and show maximum extent of sprouting area. (**B-C**) Quantification of vascular sprouting at Day 8 of CSA. Choroid sprouting is significantly increased with 48/80 treatment compared with control (HBSS) treatment. Sprouting curve shown from Day 0 to 10. (**D-E**) Western blot reveal 48/80-induced increased expression of TGF-B in CA supernatant. (**D**) Representative western blot of TGF-B. (**E**) Densitometric quantification of TGF-B western blot. (**F**) Representative images showing decreased sprouting caused by KF. (**G-H)** Quantification of vascular sprouting at Day 8 of CSA. Choroid sprouting is significantly decreased in KF + 48/80 treatment compared with only 48/80 treatment. Sprouting curve shown from Day 0 to 8. (**I)** Representative images showing decreased sprouting caused by VTI-1002 despite mast cell activation. (**J-K**) Quantification of sprouting at Day 8 of CSA. Choroidal sprouting is significantly decreased in VTI-1002 + 48/80 treatment compared with only 48/80. Sprouting curve shown from Day 0 to 8. Results are presented as mean ‡ SEM. *p < 0.05 in T-test. n of **A-C**, = 4 per group, n of **D-E** and **F–H** = 3 per group. 6600 pixels = 1mm^2^ sprouting. Scale bar = 1 mm

Exogenous treatment using the mast cell activator, 48/80, resulted in increased growth in the CSA. Figure 9A and B shows representative pictures of explants treated with HBSS (control) or 48/80, and on Day 8, there is significantly more sprouting in the 48/80 group (p < 0.005). This difference appears on Day 4 and remained until Day 10 (Fig. 9C). To ensure mast cell degranulation occurred after 48/80 treatment, some explants were removed, bleached, and stained with toluidine blue. The 48/80 treated explants contained degranulated mast cells while control tissues did not (Supplementary Fig. 2).

Western blot analysis after 48/80 treatment revealed an increase in TGF-B in the CSA supernatant, similar to what was observed after exogenous GzmB application to CSA explants. Densiometric analysis of the WB revealed a significant increase in TGF-B content in 48/80 treated explants compared to controls (Fig. 9D, E). Interestingly, drusen deposits contain inflammatory mediators, complement factors such as C5a and C3a, which can activate mast cells [42, 48, 49]. While mast cell degranulation caused increased choroidal sprouting (Fig. 9A–C), we also investigated the impact of stabilizing mast cells in the presence of an activator. After treatment with ketotifen fumarate (KF), a mast cell stabilizer, applied in conjunction with 48/80, we saw a decrease in the choroidal sprouting response starting from Day 4 as shown in Fig. 9H (p < 0.01) [41, 50]. The representative picture and graph from Day 8 (Fig. 9F–H) of the sprouting indicate a drastic reduction in sprouting. A similar reduction in neovascularization has been reported in CNV experiments where mast cells were stabilized [50].

Given that increased sprouting was evident in CSAs treated with exogenous GzmB (Fig. 2A, B), and again with 48/80, a degranulation agent, we hypothesized that the GzmB, or other proteases such as tryptase, released from mast cells may be a significant contributor to the neovascularization. While there are no known specific inhibitors of tryptase, there is a specific inhibitor of GzmB. Therefore, we next inhibited mast cell-derived GzmB by application of VTI-1002, a small molecule, specific inhibitor against GzmB (Fig. 9I) [51]. After Day 6, there was a significant decrease in the extent of sprouting from the 48/80 + VTI-1002 treated explants compared to the 48/80 only group (Fig. 9K). This difference was maintained into Day 8, where the 48/80 treated explants continue to sprout more compared to the 48/80 + VTI-1002 treated explants (Fig. 9J). Notably, the difference between the 48/80 + VTI-1002 explants and the VTI-1002 treated explants was not significant (Fig. 9J). These results suggest that other mast cell components, such as tryptase, may not have as significant of an effect as GzmB on the sprouting in the choroidal explants. Additional experiments in which we can specifically inhibit tryptase will allow us to further validate the role of these other proteases in choroidal neovascularization.

## Discussion

This study identifies GzmB, a here-to-fore unknown ocular serine protease, as a potential new target to suppress choroidal neovascularization. Novel strategies, such as targeting GzmB, have potential to 1) suppress the remodeling of the BrM and thereby maintain the sequestration of angiogenic factors such as VEGF, and 2) slow the cleavage of the RPE tight junctional proteins and thereby preserve the integrity of the outer blood-retinal barrier. Furthermore, we suggest that inhibition of extracellular GzmB activity may be an adjunctive therapy to support existing anti-VEGF treatments, and to potentially treat AMD patients who are non-responsive or whose disease has become resistant to anti-VEGFs drugs.

GzmB is present in RPE and choroidal mast cells in mouse, human and, as revealed in this study, non-human primate [15, 23]. Using a mouse choroidal sprouting assay, exogenous application of GzmB promoted choroidal angiogenesis and inflammation, through ECM remodelling, pro-inflammatory and pro-angiogenesis pathways. These data support the potential therapeutic efficacy of inhibiting extracellular GzmB for wet AMD, as pharmacological inhibition of GzmB after mast-cell degranulation significantly reduces choroidal angiogenesis. Furthermore, we observed a reduction in CNV and inflammation in GzmB-deficient mice, and an amelioration of age-related deterioration of ERG responses compared to wild-type controls.

The major risk factor for AMD is aging, and our data here as well as our earlier studies support an age-related accumulation of extracellular GzmB in the outer retina and choroid of older nonhuman primates and older human eyes; these findings confirm that the immunoreactivity for GzmB increases with age, thereby an important potential age-related factor in the pathogenesis of AMD [15, 23]. The localization of GzmB in RPE cells near drusen sites, a hallmark of AMD, further supports this concept [42]. These results provide a foundation for future studies in nonhuman primates, arguably the best animal model for studying novel treatment strategies for AMD patients.

### GzmB promotes extracellular matrix degradation

Bruch's membrane (BrM) is a crucial ECM layer that separates the RPE and choriocapillaris, providing structural support and regulating various pathways that are important for retinal health [17–19]. However, remodeling of the BrM via the degradation of its ECM proteins can lead to increased vascular permeability and pro-angiogenic responses, ultimately leading to cell death [52–55]. Our study shows the capability of exogenous GzmB to significantly degrade the ECM proteins fibronectin, laminin and decorin in the RPE-choroid, indicating that GzmB may play a role in the ECM remodeling that occurs in AMD. GzmB-induced cleavage of important ECM proteins in the BrM may diminish the outer retinal barrier function, promote vascular leakage and inflammation, and dysregulate angiogenesis in CNV observed in nAMD. Decorin, a substrate of GzmB and an important proteoglycan within the choroidal ECM, is an anti-angiogenic molecule, which when cleaved by GzmB loses its anti-angiogenic activity [56]. This result is consistent with our previous findings and others’ research showing GzmB degradation of ECM proteins including decorin (thereby releasing TGF-β) [57], fibronectin and laminin [15, 58, 59], causing further degradation of the BrM. We have also previously shown that GzmB contributes to vascular permeability via the proteolytic release of VEGF that is sequestered in the ECM [60].

GzmB also induces detachment-mediated cell death, further exacerbating inflammation via immune responses [54, 61–65]. Our previous study [66] and other studies [67, 68] also support that the accumulation of fibronectin fragments (FN-fs) from GzmB-induced degradation can supplement the release of inflammatory cytokines, matrix metalloproteinases (MMPs), endothelial cell proliferation, and monocyte chemoattractant proteins (MCPs) to sustain chronic inflammation in the RPE-choroid.

### GzmB induces inflammation

Chronic inflammation is a key contributing factor in the pathogenesis of AMD, and increased expression of proinflammatory cytokines including IL-6, IL-8 and TGF-β have been observed in the disease [35, 69–72]. Investigating the mechanism of chronic inflammation in AMD is crucial to its treatment. Our data shows that exogenous GzmB can promote inflammation and monocyte chemotaxis in the RPE-choroid by increasing the expression of proinflammatory cytokines IL-6 and TGF-β, as well as the chemokine CCL2. In naïve GzmB−/− mice, GzmB deficiency leads to a decrease in the number of perivascular macrophages in the outer retina, suggesting that GzmB plays a crucial role in the initiation of proinflammatory pathways that contribute to the development of CNV. Furthermore, mice with GzmB deficiency exhibited less IL-6 immunofluorescence and fewer microglial cells within CNV lesions after laser induction compared to age-matched WT. Previously, we have shown that GzmB induces IL-8 expression and macrophage inflammatory protein-2 (MIP-2), increasing neutrophil chemotaxis, and potentially monocyte chemotaxis in pemphigoid diseases [35], and also releasing decorin-sequestered active TGF-β [57]. Overall, our findings are consistent with previous research linking GzmB to chronic inflammation in various pathologies, including nAMD, cardiovascular, pulmonary, and skin disorders (Reviewed in Dubchak et al., 2022 [23] and Jung et al., 2022 [73]).

VEGF-A levels play a crucial role in the development of CNV in AMD, and TSP-1 is known to act as a potent inhibitor of angiogenesis [74, 75]. We show that exogenous GzmB promotes choroidal vascular sprouting, increases VEGF-A expression, and reduces TSP-1 levels, providing a mechanism for the proangiogenic effects of GzmB in the RPE-choroid complex. In a recent work [76], we showed that TSP-1 is a substrate of GzmB and thereby promotes CNV by two means, one in which sequestered VEGF is released from the ECM, and another by the degradation of TSP-1, a known anti-angiogenic factor produced by RPE and present in choroidal tissues. In naïve GzmB−/− mice, our findings also demonstrate that GzmB deficiency leads to a reduction in VEGF-A. Also, in the laser-induced CNV model, we demonstrated that GzmB deficiency significantly suppresses CNV development as represented by reduced CD31 and VEGF expression, and reduced CNV lesion sizes. Together, these findings provide evidence for the role of GzmB in the development of CNV, and the findings are in line with previous studies that have shown the involvement of GzmB in the proangiogenic process in various chronic pathological conditions (Reviewed in Dubchak et al., 2022 [23] and Jung et al., 2022 [73]). Furthermore, it is important to note that GzmB-induced proangiogenesis via a VEGF-A/TSP1 pathway in the RPE-choroid complex may contribute to the development of nAMD.

### Visual function is ameliorated in Aged GzmB−/− mice

ERG a waves and b-waves are important indicators of visual function in AMD, as they provide a non-invasive measure of retinal electrical response to light stimuli, thereby assessing retinal function and can evaluate changes in the retina that occur with aging and disease progression [36]. Melanin in the RPE is a major ocular absorber of light, and a decrease in RPE melanin is a sign of normal ocular aging and an AMD symptom. Hence, measuring the concentration of melanin in the RPE can provide assessments of the development and progression of AMD [77, 78]. Our study provides evidence that GzmB deficiency rescues age-related loss of visual function as measured by ERG responses (a-wave and b-wave amplitudes) and restores melanin content and contrast in the outer retina. These findings are significant in the context of the role of GzmB in AMD as they suggest that the absence of GzmB may protect against age-related deterioration in visual function and melanin content and contrast in the outer retina.

Functional in vivo imaging, which was previously developed from a swept source-based small animal retinal imaging system [25], allowed us to visualize with better resolution and high phase stability. Melanin content, imaged with polarization diversity detection of melanin-specific contrast imaging allowed us to identify that the CNV lesions in WT contain more widespread melanin disruption compared to GzmB−/− [26, 27]. The OCT-based angiography (OCTA) images were derived from three consecutive OCT scanning at a single transversal location to visualize retinal vasculature and demonstrated more choroidal blood flow in WT compared to GzmB−/− [25, 28, 29].

### Choroidal mast cell degranulation promotes CNV

Recent evidence suggests that the degranulation of mast cells may contribute to the pathogenesis of AMD including nAMD and geographic atrophy (GA), by causing the death of the RPE, choriocapillaris and the development of CNV and GA [40–42, 50, 79]. Furthermore, in a tumor model, Wroblewski et al. [80] demonstrated that mast cell degranulation, and specifically GzmB released from mast cells, promotes angiogenesis by decreasing the efficacy of anti-angiogenic agents in a tumor model. We previously showed that choroidal mast cells are a major source of GzmB in the human choroid [15], but there is scarcity of information about choroidal mast cells in mouse models which represents the majority of experimental AMD models. Here, we confirmed the presence of GzmB-expressing mast cells in the mouse and human choroid/sclera and our characterization of these GzmB-expressing choroidal mast cells show a granular appearance with GzmB-expressing choroidal mast cells in the mouse choroid demonstrating size similarity (mostly 10-25 µm in diameter) to those seen in the human choroid. Furthermore, some of the GzmB-expressing mast cells showed an active degranulation immunophenotype with GzmB-release into the choroidal extracellular space while some of the human GzmB-expressing choroidal cells were preferably localized near blood vessels. These findings primarily provide further insight into mast cells as the key source of GzmB in the choroidal space and their potential to degranulate and release GzmB extracellularly to contribute to the development of nAMD.

Our data provide compelling evidence that pharmacological inhibition of mast cell degranulation can effectively minimize choroidal sprouting. Our findings indicate that exogenous 48/80 treatment degranulates mast cells, resulting in increased choroidal sprouting, which is accompanied by an increase in TGF-β. Treatment with the mast cell stabilizer, ketotifen fumarate, in conjunction with 48/80 resulted in a significant reduction in choroidal sprouting, suggesting that mast cell degranulation may play a key role in the pro-angiogenic effects of mast cells in CNV as reported in previous studies [42, 50, 79, 81] and stabilizing mast cells can mitigate their pro-angiogenic effects. However, given that mast cells contain other proteases in addition to GzmB, we next used a specific GzmB inhibitor, VTI-1002 [51], with 48/80 which resulted in a significant decrease in the amount of choroidal sprouting, further highlighting the important role of GzmB in the pro-angiogenic effects of mast cells in choroidal neovascularization. These findings have important implications for the development of new therapies for AMD.

In summary, unrestrained extracellular GzmB activity in the outer retina and choroid due to mast cell degranulation may be a significant contributor to ECM remodelling, neovascularization, and chronic inflammation in nAMD. Our research provides new insights into the role of extracellular GzmB in the outer retina, and specifically in the development of CNV in nAMD, and suggests that targeting extracellular GzmB may represent a novel therapeutic approach to suppress CNV in nAMD. The lack of known endogenous (extracellular) inhibitors of GzmB indicates that pharmacological inhibition of extracellular GzmB may be a viable option for treating nAMD. Additionally, this method may be used as an alternative or adjuvant therapy for nAMD patients who do not respond to anti-VEGF treatments. However, further research is needed to investigate the safety and efficacy of GzmB inhibition in preclinical and clinical studies of nAMD.

### Supplementary Information

Below is the link to the electronic supplementary material.

Supplementary file1 (PDF 152 kb)

Supplementary file2 (PDF 104 kb)

Supplementary file3 (PDF 68 kb)

## Data Availability

The data that support this study are available from the corresponding author upon reasonable request.
