# Correction: Granzyme B degrades extracellular matrix and promotes inflammation and choroidal neovascularization

**DOI:** 10.1007/s10456-024-09919-7

**Published:** 2024-05-03

**Authors:** Gideon Obasanmi, Manjosh Uppal, Jing Z. Cui, Jeanne Xi, Myeong Jin Ju, Jun Song, Eleanor To, Siqi Li, Wania Khan, Darian Cheng, John Zhu, Lyden Irani, Isa Samad, Julie Zhu, Hyung-Suk Yoo, Alexandre Aubert, Jonathan Stoddard, Martha Neuringer, David J. Granville, Joanne A. Matsubara

**Affiliations:** 1 Department of Ophthalmology and Visual Sciences, UBC, Vancouver, BC Canada; 2 School of Biomedical Engineering, UBC, Vancouver, BC Canada; 3 International Collaboration On Repair Discoveries (ICORD), Vancouver Coastal Health Research Institute, University of British Columbia (UBC), Vancouver, BC Canada; 4 Department of Pathology and Laboratory Medicine, UBC, Vancouver, BC Canada; 5 Oregon Health & Science University (OHSU), Portland, OR USA

**Correction to: Angiogenesis** 10.1007/s10456-024-09909-9

In the original published article, Fig. 3e should have dotted red rectangle and a red arrow but have been missed. The correct version of Fig. 3 with correct informaion is provided in this correction.

**Fig. 3:**
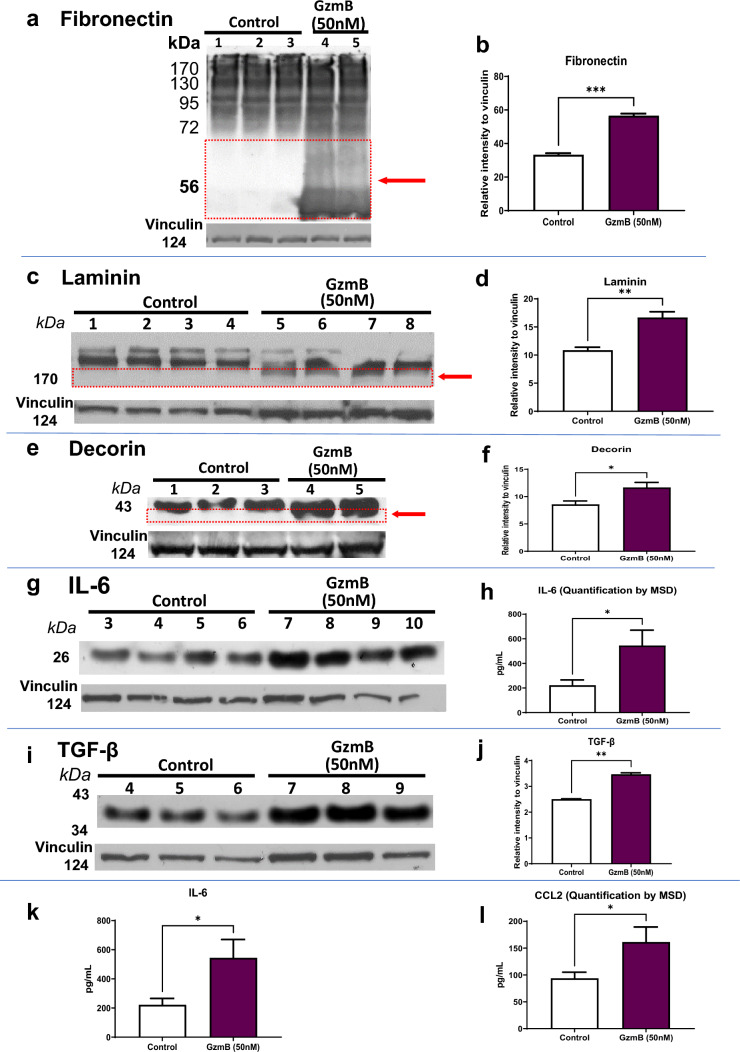
GzmB degrades the extracellular matrix and promotes inflammation in the RPE-Choroid. **A**, **C**, **E** Western blot reveals cleavage of extracellular matrix proteins by exogenous GzmB. Representative western blot of ECM proteins in CSA supernatant for **A** fibronectin; **C** laminin and **E** decorin. Note cleavage bands at lower molecular weight, identified by the red box and arrow in A (fibronectin) and C (laminin). Vinculin bands are shown as loading controls. **B**, **D**, **F** Densitometric quantification of degradation by western blot—the additional cleavage bands at lower molecular weight were quantified. **B** Fibronectin; **D** laminin and **F** decorin. Results are presented as mean ± SEM. *p < 0.05, ***p < 0.001 in T-test. n = 4 per group. **G**, **I** Next, we tested pro-inflammatory cytokines by western blot in CSA supernatant after exogenous GzmB. Representative western blot of inflammatory cytokines in CSA supernatant: **G** IL-6; **I** TGF-β. **H**, **J** Densitometric quantification of western blots. **H** IL-6; **J** TGF-β**. K**, **L** Two additional pro-inflammatory cytokines were quantified by MSD multiplex assay: **K** IL-6; **L** CCL2. Results are presented as mean ± SEM. *p < 0.05, **p < 0.01, ***p < 0.001 in T-test. n = 4–6 per group

The original article has been corrected.

