# Supplementary figures and images for: Granzyme B degrades extracellular matrix and promotes inflammation and choroidal neovascularization
Source: Angiogenesis. 2024 Mar 18;27(3):351–73. doi: 10.1007/s10456-024-09909-9 (PMC11303490; doi:10.1007/s10456-024-09909-9)

Supplementary Figure 1. Representative western blot of TSP-1

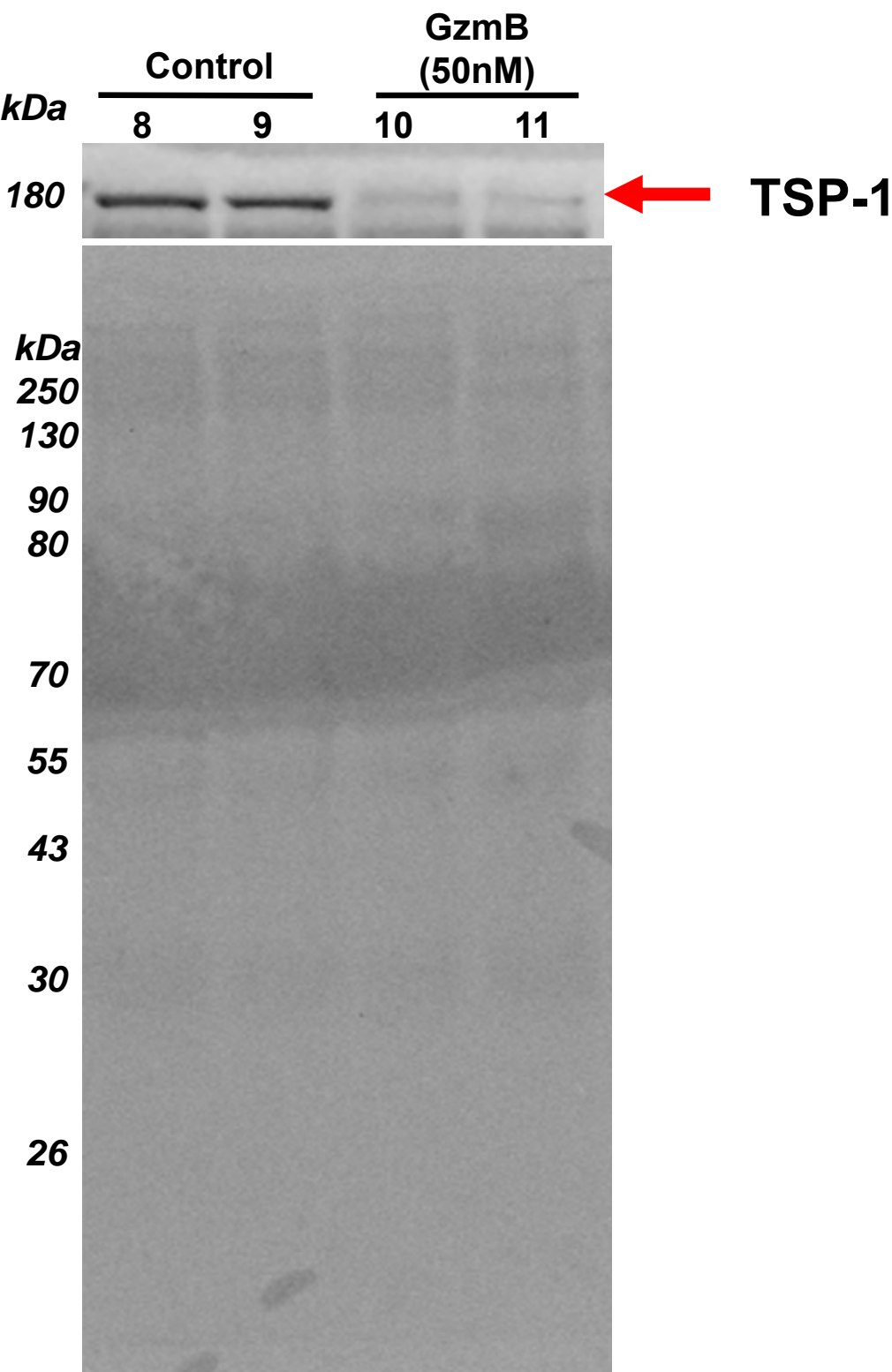

Supplement: Supplementary file 1 — Supplementary file1 (PDF 152 kb) [file 10456_2024_9909_MOESM1_ESM.pdf]
