# Supplementary material for: Granzyme B degrades extracellular matrix and promotes inflammation and choroidal neovascularization
Source: Angiogenesis. 2024 Mar 18;27(3):351–73. doi: 10.1007/s10456-024-09909-9 (PMC11303490; doi:10.1007/s10456-024-09909-9)
Supplement: Supplementary file 2 — Supplementary file2 (PDF 104 kb) [file 10456_2024_9909_MOESM2_ESM.pdf]

Supplementary Figure 2. Mast cells degranulate after 48/80

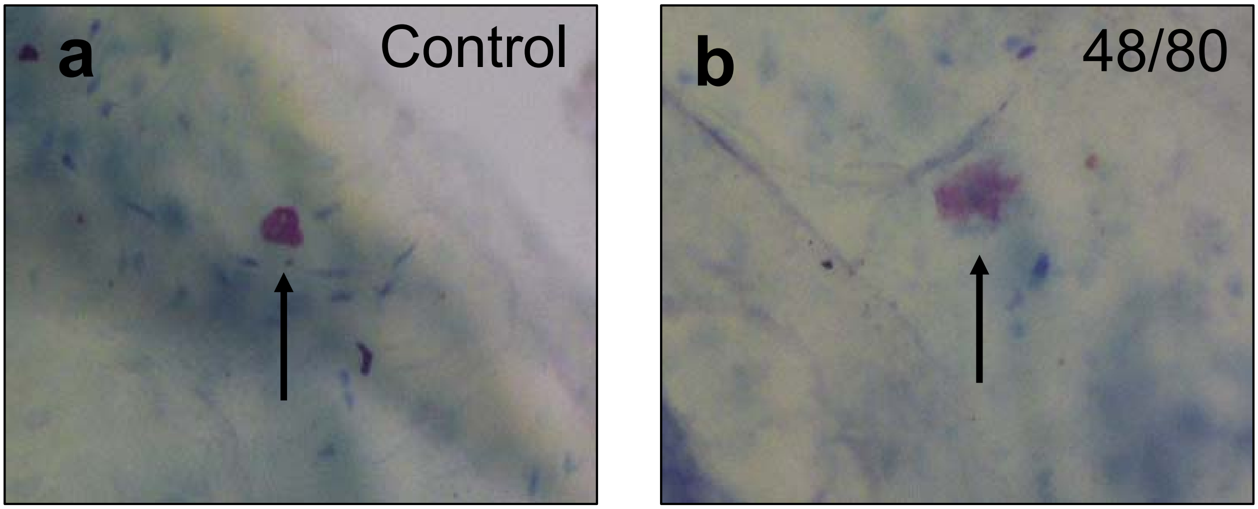

(A) Representative picture of mast cell in control (HBSS) treated bleached explant after culture in CSA. Mast cell remains condensed and circular. **(B)** Representative picture of mast cell in 48/80 treated bleached explant after culture in CSA. Mast cell activated and degranulating.
