# Supplementary material for: Granzyme B degrades extracellular matrix and promotes inflammation and choroidal neovascularization
Source: Angiogenesis. 2024 Mar 18;27(3):351–73. doi: 10.1007/s10456-024-09909-9 (PMC11303490; doi:10.1007/s10456-024-09909-9)
Supplement: Supplementary file 3 — Supplementary file3 (PDF 68 kb) [file 10456_2024_9909_MOESM3_ESM.pdf]

Supplementary Table 1. Antibody Table

Antigen	Antigen (catalog no.)	Dilution	Source
	Primary antibodies		
**GzmB**	Rabbit polyclonal anti-Granzyme B antibody (ab4059)	1:100	Abcam
**TSP-1**	Rabbit monoclonal recombinant anti-Thrombospondin 1 antibody (ab263905)	1:100	Abcam
**VEGFA**	Rabbit polyclonal -VEGFA antibody (ab46154)	1:100	Abcam
**F4/80**	Rat monoclonal anti-F4/80 antibody [CI:A3-1] – Macrophage Marker (ab6640)	1:100	Abcam
**TGF beta**	Rabbit polyclonal anti-TGF beta 1 antibody (ab92486)	1:1000	Abcam
**Fibronectin**	Rabbit polyclonal anti-Fibronectin antibody (ab2413)	1:1000	Abcam
**Laminin**	Rabbit polyclonal anti-Laminin antibody (ab11575)	1:1000	Abcam
**Decorin**	Rabbit polyclonal anti-Decorin antibody (ab175404)	1:1000	Abcam
**IL-6**	Rabbit polyclonal anti-IL-6 antibody (ab6672)	1:1000	Abcam
**CD31**	Rabbit monoclonal CD31 (PECAM-1) (D8V9E) (77699)	1:100	Cell Signaling
**c-Kit**	Rat monoclonal anti-CD117 antibody (105816)	1:100	Biolegend
	Secondary antibodies		
**biotinylated goat anti-rabbit**	biotinylated anti-rabbit made in goat secondary antibody (BA-1000)	1:200	MJS Biolynx
**Alexa 546 (Goat anti-mouse)**	Goat Anti-mouse Cy3 Alexa 546 IgG1 secondary antibody (A21123)	1:400	Fisher Scientific
**Alexa 488 (Goat anti-rabbit)**	Goat Anti-rabbit Alexa 488 secondary antibody (A11070)	1:500	Fisher Scientific
**Alexa 546 (Donkey anti-rabbit)**	Donkey anti-rabbit Alexa 546 secondary antibody (A11056)	1:500	Fisher Scientific
**Alexa 488 (Goat anti-rat)**	Goat anti-Rat IgG2b secondary antibody (FITC) (NB7129)	1:200	Novus Biologicals
